# Ising superconductivity induced from spin-selective valley symmetry breaking in twisted trilayer graphene

**DOI:** 10.1038/s41467-023-38250-w

**Published:** 2023-05-12

**Authors:** J. González, T. Stauber

**Affiliations:** 1 Instituto de Estructura de la Materia, CSIC, E-28006 Madrid, Spain; 2 Instituto de Ciencia de Materiales de Madrid, CSIC, E-28049 Madrid, Spain

**Keywords:** Superconducting properties and materials, Electronic properties and devices

## Abstract

We show that the *e*-*e* interaction induces a strong breakdown of valley symmetry for each spin channel in twisted trilayer graphene, leading to a ground state where the two spin projections have opposite sign of the valley symmetry breaking order parameter. This leads to a spin-valley locking in which the electrons of a Cooper pair are forced to live on different Fermi lines attached to opposite valleys. Furthermore, we find an effective intrinsic spin-orbit coupling explaining the protection of the superconductivity against in-plane magnetic fields. The effect of spin-selective valley symmetry breaking is validated as it reproduces the experimental observation of the reset of the Hall density at 2-hole doping. It also implies a breakdown of the symmetry of the bands from *C*_6_ to *C*_3_, with an enhancement of the anisotropy of the Fermi lines which is at the origin of a Kohn-Luttinger (pairing) instability. The isotropy of the bands is gradually recovered, however, when the Fermi level approaches the bottom of the second valence band, explaining why the superconductivity fades away in the doping range beyond 3 holes per moiré unit cell in twisted trilayer graphene.

## Abstract (web summary)

Superconductivity has been observed experimentally in twisted trilayer graphene. Here, the authors show theoretically that extended electron-electron Coulomb interaction induces breakdown of spin-selective valley symmetry in twisted trilayer graphene, which leads to Ising superconductivity.

## Introduction

The discovery of superconductivity and its parent insulating phases at the magic angle of twisted bilayer graphene (TBG)^1, 2^ has opened a new era in the investigation of strongly correlated phenomena in two-dimensional electron systems. There is an ongoing debate about the origin of the superconductivity in TBG^3–40^, which could also clarify whether a similar phenomenon can arise in other moiré van der Waals materials. In this regard, superconductivity has been already observed in twisted trilayer graphene (TTG)^41,42^, showing unconventional features like reentrant behavior under large magnetic fields^43–49^. Moreover, in the presence of spin-orbit coupling, a valley symmetry (VS) broken state can lead to a zero-field superconducting diode effect^50,51^.

TTG has also shown a striking phenomenon of reset of the Hall density at integer fillings of the highest valence and lowest conduction bands^41,42^. Specifically at 2-hole doping, it has been found that the Hall density jumps down to zero. This observation is particularly important, since the effect of reset precedes the development of the superconducting regime right below 2-hole doping as well as right above 2-electron doping in the conduction band.

**Fig. 1:**
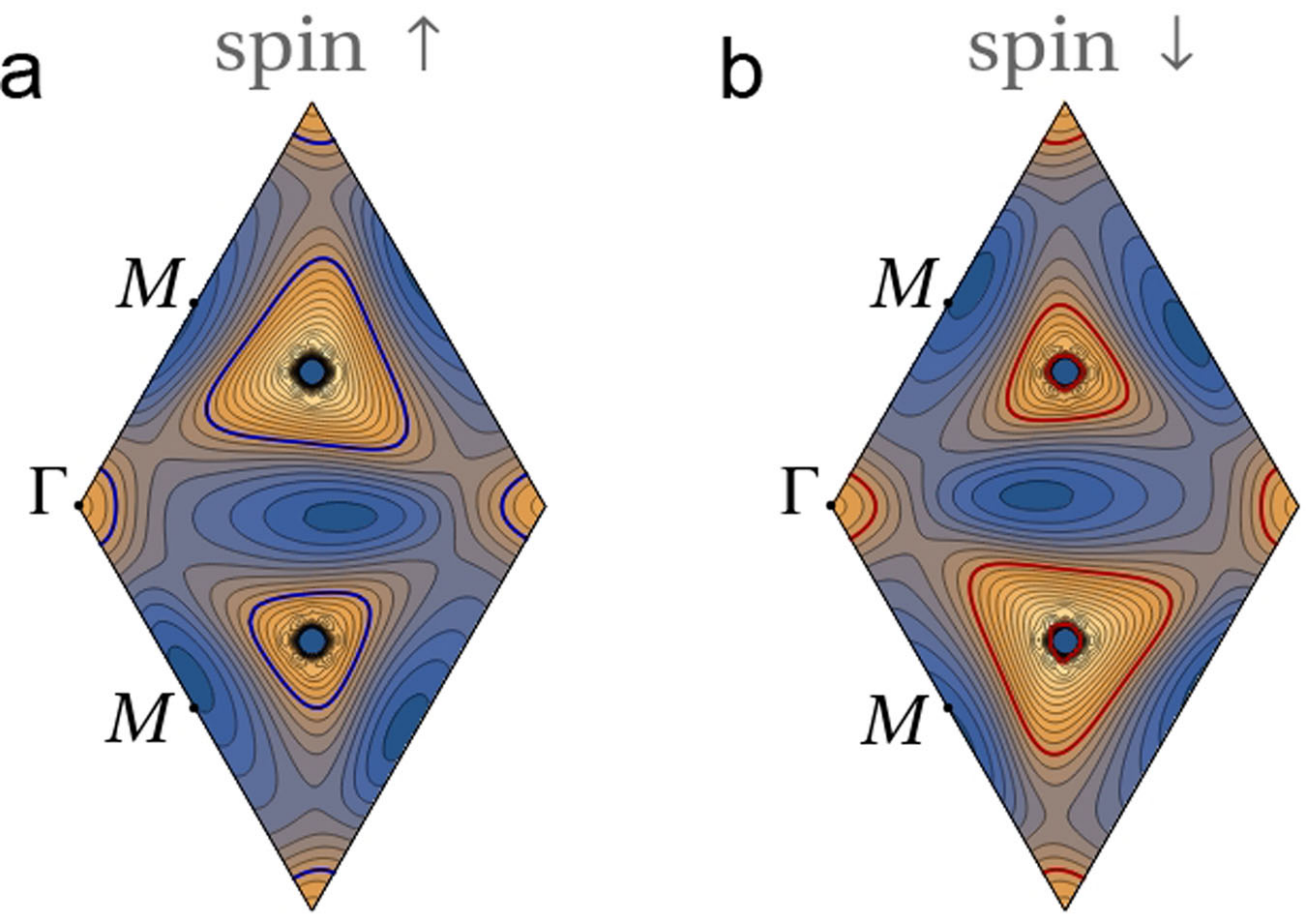
Energy contour maps of the second valence band at filling fraction *ν* = −2.4. **a** Fermi lines for spin-up electrons. **b** Fermi lines for spin-down electrons. Energy contours are shown on the moiré Brillouin zone of TTG with twist angle *θ* ≈ 1.61^∘^, for dielectric constant *ϵ* = 48 and filling fraction of 2.4 holes per moiré unit cell. Contiguous contour lines differ by a constant step of 0.2 meV.

**Fig. 2:**
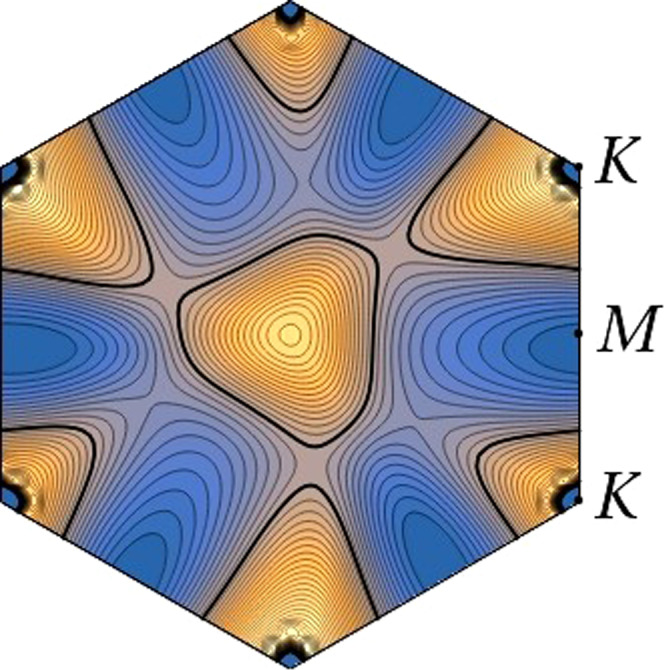
Energy contour map of the second valence band at filling fraction *ν* = −2.8. Energy contour map of the second valence band (for spin-up projection) in the Brillouin zone of TTG at twist angle *θ* ≈ 1.61^∘^, computed in a self-consistent Hartree-Fock approximation with dielectric constant *ϵ* = 48 and filling fraction of 2.8 holes per moiré unit cell. The thick contour stands for the Fermi line and contiguous contour lines differ by a constant step of 0.1 meV.

Here, we show within a self-consistent Hartree-Fock resolution in real space that the extended Coulomb interaction has a natural tendency to induce the breakdown of the VS of TTG. This lifts the degeneracy of the Dirac cones by moving them up and down in energy, respectively. The effect becomes strongest at 2-hole doping such that the Fermi level is pushed up to the vertices of the Dirac cones in the lower valley. At that filling, the Dirac nodes turn out to be unstable against time-reversal symmetry breaking with condensation of a Haldane mass, opening a gap at the Fermi level. As we show below, this is the mechanism responsible for the experimentally observed reset of the Hall density. We also show that the Fermi lines for spin-up and spin-down electrons are different but related by inversion symmetry, i.e., by the exchange of the two *K* points in the Brillouin zone, as seen in Fig. 1. However, within one spin-channel, VS breaking leads to inversion breaking, as seen in Fig. 2. Ultimately, this can explain the violation of the Pauli limit by a factor of 2–3, observed in experiments.

## Results

### Spin-selective valley symmetry breaking

We deal with the setup of TTG usually realized in the experiments, in which the two outer layers are rotated by the same angle *θ* with respect to the central layer. We model this configuration by taking a twist angle *θ* ≈ 1.61^∘^ belonging to the set of commensurate superlattices realized by TBG. Then, the low-energy states are distributed into a Dirac-like band, with states odd under mirror symmetry with respect to the central plane, and two additional valence and conduction bands, with states even under the mirror symmetry (see the Supplemental Material (SM)). The latter are the counterpart of the flat bands of TBG, and they become progressively flatter when approaching the magic angle of TTG, which is ≈1.6^∘^.

In what follows, we apply an atomistic approach to TTG, based on a tight-binding model for the *π* orbitals of the carbon atoms. The Hamiltonian *H* is written as

1 $$H={H}_{0}+{H}_{{{{{{{{\rm{int}}}}}}}}},$$

where *H*_0_ stands for the non-interacting tight-binding Hamiltonian and *H*_int_ is the interaction part. This is expressed in terms of creation (annihilation) operators ${a}_{i\sigma }^{+}$ (*a*_*iσ*_) for electrons at each carbon site *i* with spin *σ*

2 $${H}_{{{{{{{{\rm{int}}}}}}}}}=\frac{1}{2}\mathop{\sum}\limits_{i,j,\sigma,{\sigma }^{{\prime} }}{a}_{i\sigma }^{{{{\dagger}}} }{a}_{i\sigma }{v}_{\sigma {\sigma }^{{\prime} }}({{{{{{{{\bf{r}}}}}}}}}_{i}-{{{{{{{{\bf{r}}}}}}}}}_{j}){a}_{j{\sigma }^{{\prime} }}^{{{{\dagger}}} }{a}_{j{\sigma }^{{\prime} }},$$

For **r**_*i*_ ≠ **r**_*j*_, we take ${v}_{\sigma {\sigma }^{{\prime} }}({{{{{{{{\bf{r}}}}}}}}}_{i}-{{{{{{{{\bf{r}}}}}}}}}_{j})=v({{{{{{{{\bf{r}}}}}}}}}_{i}-{{{{{{{{\bf{r}}}}}}}}}_{j})$, *v* being the extended Coulomb potential with the long-range tail cut-off at a distance dictated by the screening length *ξ*, arising from the presence of nearby metallic gates, and with the strength further reduced by a dielectric constant *ϵ*. For **r**_*i*_ = **r**_*j*_, we have the Hubbard interaction ${v}_{\sigma {\sigma }^{{\prime} }}=U{\delta }_{\sigma,-{\sigma }^{{\prime} }}$, where we take *U* = 0.5 eV. The precise value of this rather small coupling is not relevant, as long as it is nonvanishing, but it plays a very important role to constrain the relative orientation of the spin projections in the two valleys of TTG (see the SM for all the details about the interaction).

We resort to a self-consistent Hartree-Fock approximation in order to study the effects of the *e*-*e* interaction. In this approach, the full electron propagator *G* is represented in terms of a set of eigenvalues *ε*_*aσ*_ and eigenvectors *ϕ*_*aσ*_(**r**_*i*_) modified by the interaction, in such a way that in the static limit

3 $${\left(G\right)}_{i\sigma,j\sigma }=-\mathop{\sum}\limits_{a}\frac{1}{{\varepsilon }_{a\sigma }}{\phi }_{a\sigma }({{{{{{{{\bf{r}}}}}}}}}_{i}){\phi }_{a\sigma }^{*}({{{{{{{{\bf{r}}}}}}}}}_{j}).$$

We seek then the self-consistent resolution of the Dyson equation involving *G*, the noninteracting propagator *G*_0_ and the self-energy Σ

4 $${G}^{-1}={G}_{0}^{-1}-\Sigma .$$

The self-consistent approach becomes feasible as the electron self-energy Σ is expressed entirely in terms of the set of *ϕ*_*aσ*_(**r**_*i*_). In the static limit, we have

5 $${\left(\Sigma \right)}_{i\sigma,j\sigma }=	 {{\mathbb{I}}}_{ij}{\mathop{\sum}\limits_{a}}^{{\prime} }\mathop{\sum}\limits_{l,{\sigma }^{{\prime} }}{v}_{\sigma {\sigma }^{{\prime} }}({{{{{{{{\bf{r}}}}}}}}}_{i}-{{{{{{{{\bf{r}}}}}}}}}_{l}){\left|{\phi }_{a{\sigma }^{{\prime} }}({{{{{{{{\bf{r}}}}}}}}}_{l})\right|}^{2}\\ 	-{v}_{\sigma \sigma }({{{{{{{{\bf{r}}}}}}}}}_{i}-{{{{{{{{\bf{r}}}}}}}}}_{j}){\mathop{\sum}\limits_{a}}^{{\prime} }{\phi }_{a\sigma }({{{{{{{{\bf{r}}}}}}}}}_{i}){\phi }_{a\sigma }^{*}({{{{{{{{\bf{r}}}}}}}}}_{j}),$$

where the prime means that the sum is to be carried over the occupied levels^52^.

The Fock contribution in Eq. (5) becomes essential in order to account for the dynamical symmetry breaking. In TTG, we find that the dominant patterns correspond to the breakdown of time-reversal invariance. This may be characterized by two different order parameters

6 $${P}_{\pm }^{(\sigma )}={{{{{{{\rm{Im}}}}}}}}\left(\mathop{\sum}\limits_{i\in A}{\left({h}_{{i}_{1}{i}_{2}}^{(\sigma )}{h}_{{i}_{2}{i}_{3}}^{(\sigma )}{h}_{{i}_{3}{i}_{1}}^{(\sigma )}\right)}^{\frac{1}{3}}\pm \mathop{\sum}\limits_{i\in B}{\left({h}_{{i}_{1}{i}_{2}}^{(\sigma )}{h}_{{i}_{2}{i}_{3}}^{(\sigma )}{h}_{{i}_{3}{i}_{1}}^{(\sigma )}\right)}^{\frac{1}{3}}\right)$$

where the sums run over the loops made of three nearest neighbors *i*_1_, *i*_2_ and *i*_3_ of each atom *i* in graphene sublattices *A* and *B*, with matrix elements

7 $${h}_{ij}^{(\sigma )}={\mathop{\sum}\limits_{a}}^{{\prime} }{\phi }_{a\sigma }({{{{{{{{\boldsymbol{r}}}}}}}}}_{i}){\phi }_{a\sigma }^{*}({{{{{{{{\boldsymbol{r}}}}}}}}}_{j}),$$

which can be interpreted as an effective hopping between sites *i* and *j*. One can check that ${P}_{-}^{(\sigma )}$ gives a measure of the mismatch in the energy shift of the bands in the two valleys of the electron system. On the other hand, a nonvanishing ${P}_{+}^{(\sigma )}$ is the hallmark of a Chern insulating phase, as described originally by Haldane^53^.

**Fig. 3:**
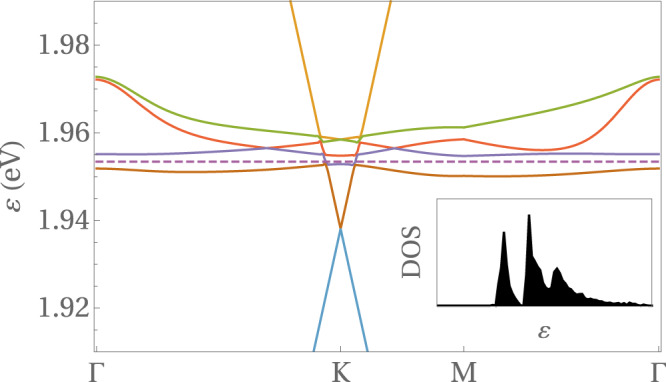
Self-consistent band structure along high-symmetry lines at filling fraction *ν* = −2. Highest valence and lowest conduction bands of TTG at twist angle *θ* ≈ 1.61^∘^, computed in a self-consistent Hartree-Fock approximation with dielectric constant *ϵ* = 48 and filling fraction of 2 holes per moiré unit cell (the dashed line stands for the Fermi level). The inset shows the density of states in the energy interval between 1.94 eV and 1.98 eV.

The analysis of internal screening in TTG reveals that the effective value of the dielectric constant must have in our model a magnitude of *ϵ* ~ 50 (see SM). The extended Coulomb interaction is then in a regime where the dominant order parameter is that of VS breaking, while ${P}_{+}^{(\sigma )}$ becomes also nonvanishing at filling fraction *ν* = − 2. This can be seen in Fig. 3, which shows the splitting at the *K* point of the Dirac cones from the two valleys, as an effect of VS breaking. At 2-hole doping, the Fermi level should be then at the vertex of the Dirac cone of the lower valley. However, the interaction is strong enough to trigger the condensation of the Haldane mass, which leads to the gap seen in Fig. 3 at the Fermi level. In this discussion, the effect of the “third”, lowest Dirac cone can be safely neglected as this band belongs to a different representation of the mirror symmetry.

### Hall density reset

From the resistivity tensor *ρ* as function of the magnetic field *B*, the Hall density *n*_*H*_ can be obtained which is usually directly related to the electronic density *n*:

8 $${n}_{H}=-{\left[e\frac{d{\rho }_{xy}}{dB}\right]}^{-1}$$

Experimentally, a reset from a large value down to zero Hall density is observed in TTG at 2-hole doping (as well as at 2-electron doping in the conduction side). In our interacting model, we can explain such a discontinuity as a result of the jump of the Fermi level across the gap shown in Fig. 3, from the bottom of the first valence band (VB) to the top of the second VB.

As shown in the SM, in the semiclassical approximation, closed trajectories quite generally lead to a universal Hall density *n*_*H*_ = *n*, in terms of the electronic density *n*. Even extreme elliptic trajectories still fall under this universality class and anharmonic effects due to trigonal warping usually lead only to slight deviations. Thus, linear (universal) behavior *n*_*H*_ = *n* is obtained starting from filling factor *ν* = 0.

Non-universal behavior with *n*_*H*_ ≠ *n* only comes from open trajectories which are usually linked to van Hove singularities (vHSs)^54^. Around these points, the diverging Hall density is given by

9 $${n}_{H}=\frac{n}{\pi }\ln \frac{\alpha {\Lambda }^{2}}{|\mu |+{k}_{B}T},$$

where *α* is related to the inverse reduced mass, Λ is the phenomenological band-cutoff, and *μ* the relative chemical potential corresponding to the electronic density *n*. We also introduce the finite temperature *T* that smears out the logarithmic divergence, which shall also include disorder effects. Details on the derivation of Eq. (9) and the fitting procedure are given in the SM.

For a quantitative discussion of the Hall density in TTG, we consider the first and second VBs for *ν* = −2 and *ν* = −2.8, respectively, see SM. We expect deviations due to varying filling factors to only slightly shift the energy of the vHS corrections. Due to the pronounced gap between the first and the second VB, there is a reset of the Hall density at *ν* = −2, which leads to *n*_*H*_ = *ν* + 2 for *ν* < −2 due to the closed semi-classical orbits of the band structure near the band edge. As mentioned above, the linear (universal) behavior is also obtained around filling factors *ν* = 0 and *ν* = −4 (neglecting the contribution of the Dirac cone that becomes relevant for *ν* ≈ −4).

**Fig. 4:**
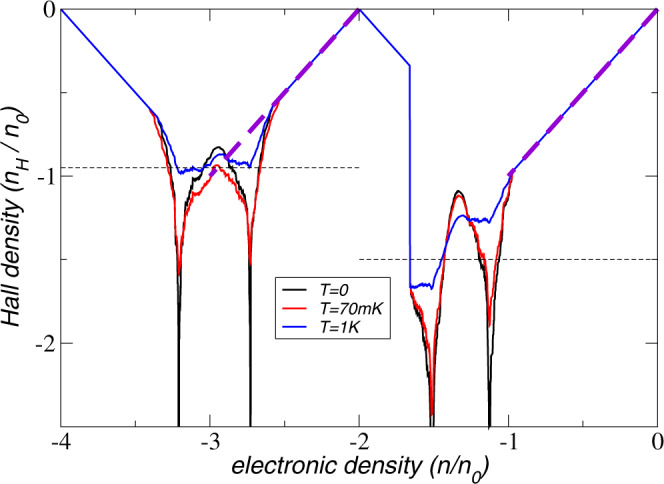
Hall density for the two highest valence bands. Hall density as function of the filling factor in units of the density *n*_0_ of one electron per moiré supercell for three different temperatures *T* = 0, 70 mK, 1K. Also shown are the maximal values for each sub-band of the Hall density measured in Ref. ^42^, as well as the dashed purple lines indicating the universal behavior. The reset at 2-hole doping emerges due to the gap at the half-filled VB, see Fig. 3.

Figure 4 shows the Hall density *n*_*H*_ as function of the filling factor for different temperatures *T* = 0, 70 mK, 1 K. The energies and respective filling factors of the vHSs are indicated by the logarithmic divergences for *T* = 0. Also shown are the maximal values for each sub-band of the Hall density measured in Ref. ^42^, as well as the dashed purple lines indicating the universal behavior. The curve for *T* = 1 K agrees well with the experimental results performed at *T* = 70 mK, which suggests a considerable amount of disorder in the unbiased sample.

### Ising superconductivity

**Fig. 5:**
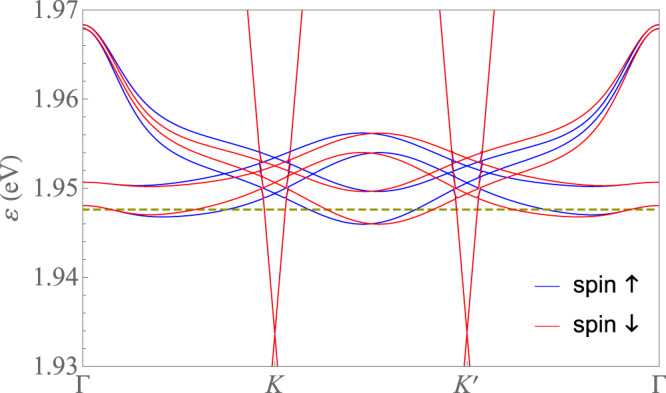
Self-consistent band structure for both spin projections along high-symmetry lines at filling fraction *ν* = −2.4. Energy bands of TTG around charge neutrality (computed for dielectric function *ϵ* = 48 and filling fraction *ν* = −2.4) along a rectilinear path $\Gamma K{K}^{{\prime} }\Gamma$, discerning the dispersion for spin-up and spin-down electrons.

The strong spin-selective VS breaking leads to ground states where the inversion symmetry is broken for each spin projection, but in which this symmetry is recovered upon exchange of the two spin projections, as shown in Fig. 5. This opens the possibility of having Ising superconductivity, in which each spin projection in a Cooper pair is attached to a different Fermi line and the singlet is polarized in out-of-plane direction^55–57^. This lends protection to the superconductivity against in-plane magnetic fields as no Zeeman term arises.

The actual pairing instability takes place as a result of the anisotropy of the *e*-*e* scattering along the Fermi lines, which is strong enough to induce an effective attraction. This is characterized by the appearance of a negative coupling when projecting the Cooper pair vertex *V* onto the different harmonics along the Fermi line. The vertex *V* is indeed a function of the angles *ϕ* and ${\phi }^{{\prime} }$ of the respective momenta of the spin-up incoming and outgoing electrons on each contour line of energy *ε*. The scattering of Cooper pairs in the particle-particle channel leads to a reduction of the amplitude of the vertex, given by the equation

10 $$V(\phi,{\phi }^{{\prime} })={V}_{0}(\phi,{\phi }^{{\prime} })- \frac{1}{{(2\pi )}^{2}}{\int}^{{\Lambda }_{0}}\frac{d\varepsilon }{\varepsilon }\int\nolimits_{0}^{2\pi }d{\phi }^{{\prime\prime} }\frac{\partial {k}_{\perp }}{\partial \varepsilon }\frac{\partial {k}_{\parallel }}{\partial {\phi }^{{\prime\prime} }}{V}_{0}(\phi,{\phi }^{{\prime\prime} })V({\phi }^{{\prime\prime} },{\phi }^{{\prime} })$$

where *k*_∥_, *k*_⊥_ are the longitudinal and transverse components of the momentum for each energy contour line while ${V}_{0}(\phi,{\phi }^{{\prime} })$ is the bare vertex at an energy cutoff Λ_0_ (see SM). By differentiating Eq. (10), we get

11 $$\varepsilon \frac{\partial \widehat{V}(\phi,{\phi }^{{\prime} })}{\partial \varepsilon }=\frac{1}{2\pi }\int\nolimits_{0}^{2\pi }d{\phi }^{{\prime\prime} }\widehat{V}(\phi,{\phi }^{{\prime\prime} })\widehat{V}({\phi }^{{\prime\prime} },{\phi }^{{\prime} })$$

with $\widehat{V}(\phi,{\phi }^{{\prime} })=F(\phi )F({\phi }^{{\prime} })V(\phi,{\phi }^{{\prime} })$ and $F(\phi )=\sqrt{(\partial {k}_{\perp }/\partial \varepsilon )(\partial {k}_{\parallel }/\partial \phi )/2\pi }$. Then, when there is a negative eigenvalue in the expansion of $\widehat{V}$ in harmonics, Eq. (11) leads to a divergent flow for that particular eigenvalue as *ε* → 0, which is the signature of the pairing instability.

The crucial point is the determination of ${V}_{0}(\phi,{\phi }^{{\prime} })$ at the upper cutoff, for which one usually takes the interaction *v* dressed at the scale Λ_0_. The relevant electron-hole processes can be summed up to give (see SM)

12 $${V}_{0}(\phi,{\phi }^{{\prime} })=\frac{{v}_{{{{{{{{\bf{k}}}}}}}}-{{{{{{{{\bf{k}}}}}}}}}^{{\prime} }}}{1+{v}_{{{{{{{{\bf{k}}}}}}}}-{{{{{{{{\bf{k}}}}}}}}}^{{\prime} }}{\chi }_{{{{{{{{\bf{k}}}}}}}}-{{{{{{{{\bf{k}}}}}}}}}^{{\prime} }}}+\frac{{v}_{{{{{{{{\boldsymbol{Q}}}}}}}}}^{2}{\widetilde{\chi }}_{{{{{{{{\bf{k}}}}}}}}+{{{{{{{{\bf{k}}}}}}}}}^{{\prime} }}}{1-{v}_{{{{{{{{\boldsymbol{Q}}}}}}}}}{\widetilde{\chi }}_{{{{{{{{\bf{k}}}}}}}}+{{{{{{{{\bf{k}}}}}}}}}^{{\prime} }}},$$

where ${{{{{{{\bf{k}}}}}}}},{{{{{{{{\bf{k}}}}}}}}}^{{\prime} }$ are the respective momenta for angles $\phi,{\phi }^{{\prime} }$ and ${\chi }_{{{{{{{{\bf{q}}}}}}}}},{\widetilde{\chi }}_{{{{{{{{\bf{q}}}}}}}}}$ are particle-hole susceptibilities at momentum transfer **q**, defined in the SM.

It now remains to expand the vertex *V*_0_ in the different harmonics $\cos (n\phi ),\sin (n\phi )$. We illustrate here this analysis taking in particular the dispersion of the second VB represented in Fig. 1, for filling fraction *ν* = −2.4. Similar analyses corresponding to *ν* = −2.8 and *ν* = −3.6 can be found in the SM, showing the trend of decreasing pairing strength.

The results of the expansion can be grouped in terms of irreducible representations of the symmetry group of the dispersion, as shown in Table 1 for *ν* = −2.4. We observe that there are several negative eigenvalues corresponding to different harmonics (with angles measured from one of the corners of the triangle-like Fermi lines in Fig. 1). From the resolution of Eq. (11), the dominant negative eigenvalue *λ* leads to a pole at a critical energy scale (see SM)

13 $${\varepsilon }_{c}={\Lambda }_{0}{e}^{-1/|\lambda|}$$

This can be translated into the critical temperature *T*_*c*_ of the pairing instability. At *ν* = −2.4, the Fermi level is near the middle of the second VB shown in Fig. 1, so we can take Λ_0_ as half the bandwidth (≈ 1.5 meV). Then, we estimate *T*_*c*_ ~ 1 K, which is consistent with the order of magnitude found in the experiments.

**Table 1:** Dominant eigenvalues of the Cooper-pair vertex

Eigenvalue *λ*	harmonics	Irr. Rep.
2.66	1	
1.80	{cos(*ϕ*),sin(*ϕ*)}	E
0.65	cos(3*ϕ*)	*A*_1_
0.42	{cos(4*ϕ*),sin(4*ϕ*)}	E
−0.37	{cos(4*ϕ*),sin(4*ϕ*)}	E
−0.37	sin(3*ϕ*)	*A*_2_
0.22	{cos(5*ϕ*),sin(5*ϕ*)}	E
0.18	sin(6*ϕ*)	*A*_2_

Eigenvalues of the Cooper-pair vertex with largest magnitude and dominant harmonics, grouped according to the irreducible representations of the approximate *C*_3*v*_ symmetry, for the Fermi line shown in Fig. 1. The modes $\{\cos (4\phi ),\sin (4\phi )\}$ appear twice in the list, as they only denote the dominant harmonic, but they actually represent different eigenvectors.

A detailed inspection shows that the nesting between parallel segments of the triangular Fermi lines for opposite spin projections (as seen in Fig. 1) is the effect behind the large magnitude of the negative couplings in Table 1. Once the Fermi line crosses to the other side of the vHS shown in Fig. 2 at *ν* ≈ −2.8, the triangular patches are replaced by elliptical Fermi lines. This comes with a decrease in the magnitude of the negative couplings, leading to a substantial drop of the critical temperature (see SM) which may explain why the superconductivity is suppressed in the experiments in that doping range.

Finally, we can estimate the critical magnetic field that is needed to break up the Cooper pairs. For an in-plane field, orbital effects can be neglected and the Zeeman term will usually shift the energy of the spin up and spin down dispersions by ± *μ*_*B*_*B*, respectively. This energy can be related to the pairing energy, giving rise to the Clogston-Chandrasekhar or Pauli limit *B*_*p*_ = 1.86*T*_*c*_ (in Tesla for *T*_*c*_ in Kelvin)^58,59^. However, due to the emergence of an imaginary hopping element between next-nearest in-plane neighbours, a Haldane flux arises which is opposite for the two spin-projections. There is thus a renormalized intrinsic spin-orbit coupling just as in the Kane-Mele model, leading to Cooper pair singlets which are polarized in out-of-plane direction. As a consequence, there is no Zeeman coupling arising from an in-plane magnetic field unless the field energy is larger than the characteristic effective spin-orbit gap Δ ~ 1 meV, see SI. The critical field can then be estimated as *B*_*c*_ = Δ/2*μ*_*B*_ ~ 8 T, assuming the electron *g*-factor equal to 2. For *T*_*c*_ ≈ 2 K, we thus find a violation of the Pauli limit by a factor 2–3, consistent with the experimental findings of Ref. ^47^.

## Discussion

We have shown that the *e*-*e* interaction induces a strong breakdown of spin-selective VS in TTG, with the two spin projections having opposite sign of the VS breaking order parameter. The two spin projections are preferentially attached to opposite *K* points, leading to an effect of spin-valley locking. In these conditions, the electrons with opposite momenta of a Cooper pair are forced to live on different Fermi lines attached to opposite valleys, giving rise to Ising superconductivity. We stress that in a conventional Ising superconductor such as NeSb_2_, the bare spin-orbit coupling leads to spin projections perpendicular to the plane^55–57^, whereas here, a renormalized spin-orbit coupling emerges as discussed by Kane and Mele^60^, leading to the same effect. Thus, a weak in-plane magnetic field cannot couple to the singlet of the Cooper pair which explains the violation of the Pauli limit, as observed experimentally.

The breakdown of VS in each spin channel leads also to a reduction of the symmetry of the bands from *C*_6_ to *C*_3_, as the latter is the symmetry enforced in a single valley. This enhanced anisotropy induces a strong modulation of the *e*-*e* scattering, which is able to trigger a Kohn-Luttinger (pairing) instability, driven solely by electron interactions^61,62^. The instability is here amplified by the strong nesting between the very regular triangular Fermi lines shown in Fig. 1, leading in particular to an attractive interaction in two channels corresponding to the $\sin (3\phi )$ harmonic and to the two-dimensional representation with $\{\cos (4\phi ),\sin (4\phi )\}$. This mechanism of attraction is progressively weakened, however, for filling fraction *ν* < −3 as the topology of the Fermi line changes into elliptic form (as seen around the *M* points in the plot of Fig. 2), explaining why there is a limited range of superconductivity in the hole-doped regime of TTG.

VS breaking seems to be a ubiquitous feature in many moiré systems, and it is plausible that its role in the development of superconductivity may be also important in other derivatives of graphene. In this regard, it is remarkable that superconductivity has been recently found in rhombohedral trilayer graphene^63–70^, which is another system close to an isospin instability. It would be pertinent then to reexamine the superconductivity of such systems in the light of spin-selective VS breaking, including TBG, to confirm the connection between the enhanced anisotropy and the Kohn-Luttinger pairing instability established in this paper. Moreover, it should be interesting to confront preliminary results on twisted quadrilayer graphene, which make us expect an odd-even effect where the superconducting instability should be most protected in the central layer present for odd multilayers.

## Methods

There are several Hartree-Fock studies using the continuum model for twisted bilayer^71–76^ or trilayer^77^ graphene. Here, however, we apply a self-consistent Hartree-Fock resolution in real space^78–80^, which allows us to include microscopic details such as the correct Coulomb interaction between the layers or the out-of-plane interaction. For details, see the Supplementary Information.

## Supplementary information

Supplementary Information

## Data Availability

The datasets generated and analyzed during the current study are available from the corresponding author on reasonable request.

## Code availability

The computer code used for the analysis and simulations in the current study are available from the corresponding author on reasonable request.
