# Author Correction: Ising superconductivity induced from spin-selective valley symmetry breaking in twisted trilayer graphene

**DOI:** 10.1038/s41467-023-39000-8

**Published:** 2023-06-13

**Authors:** J. González, T. Stauber

**Affiliations:** 1 Instituto de Estructura de la Materia, CSIC, E-28006 Madrid, Spain; 2 Instituto de Ciencia de Materiales de Madrid, CSIC, E-28049 Madrid, Spain

**Keywords:** Superconducting properties and materials, Electronic properties and devices

Correction to: *Nature Communications* 10.1038/s41467-023-38250-w, published online 12 May 2023

The original version of the Supplementary Information associated with this Article contained errors. The HTML has been updated to include a corrected version of the Supplementary Information. The original version of this Article contained an error in Table 1, in which several rows were partially empty.

The correct version of Table 1 is:

**Table:** 

Eigenvalue *λ*	harmonics	Irr. Rep.
2.66	1	
1.80	{cos(*ϕ*),sin(*ϕ*)}	E
0.65	cos(3*ϕ*)	*A*_1_
0.42	{cos(4*ϕ*),sin(4*ϕ*)}	E
−0.37	{cos(4*ϕ*),sin(4*ϕ*)}	E
−0.37	sin(3*ϕ*)	*A*_2_
0.22	{cos(5*ϕ*),sin(5*ϕ*)}	E
0.18	sin(6*ϕ*)	*A*_2_

which replaces the previous incorrect version:

**Table:** 

Eigenvalue *λ*	harmonics	Irr. Rep.
2.66	1	
1.80	{cos(*ϕ*),sin(*ϕ*)}	E
1.80		
0.65	cos(3*ϕ*)	*A*_1_
0.42	{cos(4*ϕ*),sin(4*ϕ*)}	E
0.42		
−0.37	{cos(4*ϕ*),sin(4*ϕ*)}	E
−0.37		
−0.37	sin(3*ϕ*)	*A*_2_
0.22	{cos(5*ϕ*),sin(5*ϕ*)}	E
0.22		
0.18	sin(6*ϕ*)	*A*_2_

This has been corrected in both the PDF and HTML versions of the Article.

## Supplementary information

Supplementary Information

