# Supplementary material for: Ising superconductivity induced from spin-selective valley symmetry breaking in twisted trilayer graphene
Source: Nat Commun. 2023 May 12;14:2746. doi: 10.1038/s41467-023-38250-w (PMC10182018; doi:10.1038/s41467-023-38250-w)
Supplement: Supplementary file 1 — Supplementary Information [file 41467_2023_38250_MOESM1_ESM.pdf]

SUPPLEMENTAL MATERIAL
Ising superconductivity induced from spin-selective valley symmetry breaking in twisted trilayer graphene

J. González[1] and T. Stauber[2]
[1] *Instituto de Estructura de la Materia, CSIC, E-28006 Madrid, Spain*
[2] *Instituto de Ciencia de Materiales de Madrid, CSIC, E-28049 Madrid, Spain*

SUPPLEMENTARY NOTE I.
TIGHT-BINDING APPROACH FOR TWISTED TRILAYER GRAPHENE

We model twisted trilayer graphene in a tight-binding approach, taking as starting point the non-interacting Hamiltonian:

$$H_0 = -\sum_{\langle i,j \rangle} t_{\parallel}(\boldsymbol{r}_i - \boldsymbol{r}_j)\,(a^{\dagger}_{i\sigma} a_{j\sigma} + h.c.) - \sum_{(i,j)} t_{\perp}(\boldsymbol{r}_i - \boldsymbol{r}_j)\,(a^{\dagger}_{i\sigma} a_{j\sigma} + h.c.)\,, \quad (1)$$

The sum over the brackets $\langle ... \rangle$ runs over pairs of atoms in the same layer, whereas the sum over the curved brackets $(...)$ runs over pairs with atoms belonging to different layers (1 to 3). $t_{\parallel}(\boldsymbol{r})$ and $t_{\perp}(\boldsymbol{r})$ are hopping matrix elements which have an exponential decay with the distance $|\boldsymbol{r}|$ between carbon atoms. A common parametrization is based on the Slater-Koster formula for the transfer integral[1]

$$-t(\mathbf{d}) = V_{pp\pi}(d)\left[1 - \left(\frac{\mathbf{d}\cdot\mathbf{e}_z}{d}\right)^2\right] + V_{pp\sigma}(d)\left(\frac{\mathbf{d}\cdot\mathbf{e}_z}{d}\right)^2 \quad (2)$$

with

$$V_{pp\pi}(d) = V^0_{pp\pi}\exp\left(-\frac{d-a_0}{r_0}\right)\,, V_{pp\sigma}(d) = V^0_{pp\sigma}\exp\left(-\frac{d-d_0}{r_0}\right)\,, \quad (3)$$

where $\mathbf{d}$ is the vector connecting the two sites, $\mathbf{e}_z$ is the unit vector in the z-direction, a_0 is the C-C distance and d_0 is the distance between layers. A typical choice of parameters is given by $V^0_{pp\pi} = -2.7$ eV, $V^0_{pp\sigma} = 0.48$ eV and $r_0 = 0.319 a_0$ [1].

In practice, we have taken the above values to carry out the analysis reported in the main text. We have chosen a configuration of twisted trilayer graphene belonging to the set of commensurate superlattices also realized by twisted bilayer graphene, with a twist angle $\theta \approx 1.61°$ (7566 atoms in the moiré unit cell) very close to the magic angle condition. At a first stage without out-of-plane relaxation, the tight-binding approach applied to this model leads to the low-energy bands shown in Fig. 1 about the charge neutrality point.

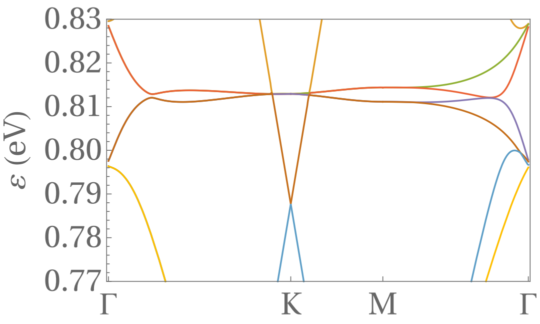

Supplementary Figure 1: Dispersion of the first valence and conduction bands about the charge neutrality point of twisted trilayer graphene with twist angle $\theta \approx 1.61°$, obtained in a tight-binding approach with no out-of-plane corrugation.

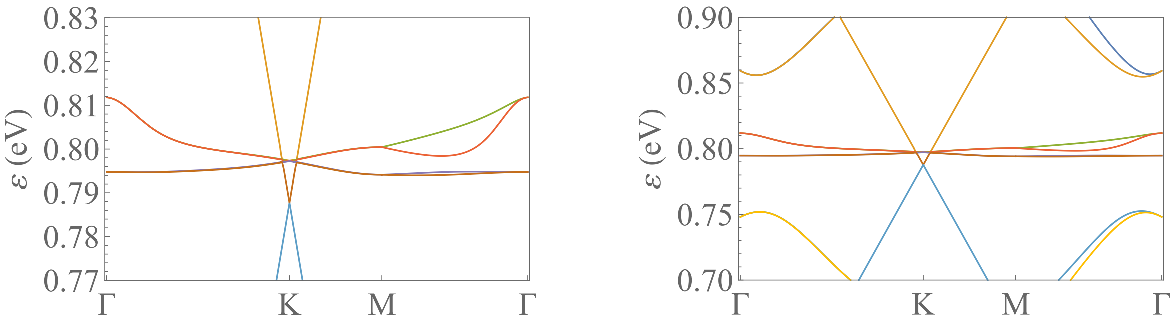

(a) (b)

Supplementary Figure 2: Dispersion of the first valence and conduction bands (zoomed out in (b)) about the charge neutrality point of twisted trilayer graphene with twist angle $\theta \approx 1.61°$, computed in a tight-binding approach with parameters given in the text and accounting for out-of-plane relaxation.

At the twist angles considered in the paper, the in-plane lattice relaxation of twisted trilayer graphene does not have the important role that it plays at the magic angle of the twisted bilayer. However, the out-of-plane corrugation of the trilayer is a relevant effect, which arises from the dependence of the interlayer interaction on the stacking of the graphene layers. Thus, the lattice structure tends to relax in the out-of-plane direction, reaching a minimum interlayer distance in the regions of AB stacking, and a maximum value in the regions of AA stacking. To describe the interlayer interaction we have used a Kolmogorov-Crespi potential[2, 3]

$$U(z) = -A\left(\frac{z_0}{z}\right)^6 + Ce^{-\lambda(z-z_0)} \tag{4}$$

where the first term stands for the van der Waals attraction and the second term accounts for an exponentially decaying repulsion due to the interlayer wave-function overlap. The effect of the registry of the carbon atoms is included in the second term, and we have adjusted it to interpolate between the different interaction energies in the regions of AB and AA stacking. In the relaxed structure we have left the central layer intact, so that the separation of the outer layers about the center becomes modulated across the superlattice according to the potential (4), reaching a minimum interlayer distance of 0.334 nm for AB stacking and a maximum distance of 0.356 nm for AA stacking.

Overall, including out-of-plane relaxation, our tight-binding approach leads to sensible results for the commensurate lattice studied in the main text with twist angle $\theta \approx 1.61°$, whose first valence and conduction bands are shown in Fig. 2.

SUPPLEMENTARY NOTE II. HARTREE-FOCK APPROXIMATION

In our microscopic approach, we may consider two different sources of electronic interaction, corresponding to the extended Coulomb interaction and the on-site (Hubbard) repulsion of electrons at the same carbon site. The first of them gives rise to a contribution to the interaction Hamiltonian which can be written in terms of creation (annihilation) operators $a^+_{i\sigma}$ ($a_{i\sigma}$) for electrons at each carbon site i with spin σ

$$H_{\rm C} = \frac{1}{2}\sum_{i,j,\sigma,\sigma'} a^\dagger_{i\sigma} a_{i\sigma}\, v(\boldsymbol{r}_i - \boldsymbol{r}_j)\, a^\dagger_{j\sigma'} a_{j\sigma'} \;, \tag{5}$$

We consider a form of the Coulomb potential v which is adapted to the case where twisted trilayer graphene is surrounded by top and bottom metallic gates. The starting point is the unscreened Coulomb potential $v_0(\boldsymbol{r}) = e^2/4\pi\epsilon r$, ϵ being the dielectric constant. In the presence of a gate at distance $z = \xi/2$, the electrostatic energy of two electrons lying in a plane parallel to the electric gate and being separated by a distance r is given by

$$v(\boldsymbol{r}) = \frac{e^2}{4\pi\epsilon}\left(\frac{1}{r} - \frac{1}{\sqrt{r^2+\xi^2}}\right) \;. \tag{6}$$

In the presence of an additional opposite gate also at distance $z = \xi/2$, and again using the image-charge technique, one obtains for the electrostatic energy[4]

$$v(\boldsymbol{r}) = \frac{e^2}{4\pi\epsilon} \sum_{n=-\infty}^{\infty} \frac{(-1)^n}{\sqrt{r^2 + n^2\xi^2}} \rightarrow \frac{e^2}{4\pi\epsilon} \frac{2\sqrt{2}\, e^{-\pi r/\xi}}{\xi\sqrt{r/\xi}} \; . \tag{7}$$

In the main text, we have used the approximate expression in Eq. (8), which is very accurate for $r/\xi \gtrsim 0.2$. We have addressed the particular case of a setup with $\xi = 10$ nm. For this screening length, the use of the expression in Eq. (8) does not modify the shape of the interacting flat bands, while determining correctly the phases and the position of the critical point for symmetry breaking and gap opening at 2-hole doping.

Moreover, we take also into account the Hubbard interaction, which can be seen as a regularization of the interaction in Eq. (5) when $\boldsymbol{r}_i = \boldsymbol{r}_j$. This leads to a contribution to the interaction Hamiltonian

$$H_{\mathrm{U}} = U \sum_i a^\dagger_{i\uparrow} a_{i\uparrow}\, a^\dagger_{i\downarrow} a_{i\downarrow} \; . \tag{8}$$

This on-site repulsion is actually the spin-dependent part of the interaction and, in this respect, it plays an important role as it helps to stabilize the iterative resolution of the self-consistent Hartree-Fock equations. For that purpose, we have taken a not too large value of the Hubbard repulsion, $U = 0.5$ eV, which is also a way of compensating the strong tendency of the Hartree-Fock approximation to overestimate the ferromagnetic instabilities arising from the spin-dependent interaction.

The self-consistent Hartree-Fock equations take then the form

$$\sum_a \varepsilon_{a\sigma}\, \phi_{a\sigma}(\boldsymbol{r}_i)\phi^*_{a\sigma}(\boldsymbol{r}_j) = \sum_a \varepsilon^0_{a\sigma}\, \phi^0_{a\sigma}(\boldsymbol{r}_i)\phi^0_{a\sigma}(\boldsymbol{r}_j) + \mathbb{I}_{ij}\, U {\sum_a}' \left|\phi_{a\,-\sigma}(\boldsymbol{r}_i)\right|^2 + \mathbb{I}_{ij} {\sum_a}' \sum_{l,\sigma'} v(\boldsymbol{r}_i - \boldsymbol{r}_l) \left|\phi_{a\sigma'}(\boldsymbol{r}_l)\right|^2 - v(\boldsymbol{r}_i - \boldsymbol{r}_j) {\sum_a}' \phi_{a\sigma}(\boldsymbol{r}_i)\phi^*_{a\sigma}(\boldsymbol{r}_j) \tag{9}$$

where $\varepsilon_{a\sigma}$ ($\varepsilon^0_{a\sigma}$) and $\phi_{a\sigma}(\boldsymbol{r}_i)$ ($\phi^0_{a\sigma}(\boldsymbol{r}_i)$) represent respectively the eigenvalues and eigenvectors building the interacting (free) electron propagator, and the prime means that the sum is to be carried over the occupied levels [5]. In our notation, $-\sigma$ represents the spin projection with opposite orientation to the spin σ.

In Eq. (9) we already see that, if the set $\{\phi_{a\sigma}(\boldsymbol{r}_i)\}$ is a self-consistent solution for a given spin projection, the set $\{\phi^*_{a\sigma'}(\boldsymbol{r}_i)\}$ is an equally good solution. If the on-site repulsion U were not present in the equations, then we could assemble a solution with $\{\phi_{a\sigma}(\boldsymbol{r}_i)\}$ and $\{\phi^*_{a\sigma'}(\boldsymbol{r}_i)\}$ corresponding to two different (arbitrary) spin orientations. The operation of complex conjugation implies the exchange of the two valleys of the twisted trilayer, so this possibility of choosing arbitrary spin orientations would mean the freedom to make independent spin rotations in each valley.

In any event, the on-site Hubbard repulsion is not vanishing, and this forces possible solutions of Eq. (9) to have opposite spin projections. When there is spin-selective valley symmetry breaking, a combined solution of the form $\{\phi_{a\sigma}(\boldsymbol{r}_i), \phi^*_{a\,-\sigma}(\boldsymbol{r}_i)\}$ corresponds then to having a nonvanishing value of the valley polarization order parameter for one of the spins, and the opposite value for the opposite spin projection. This is the basis of the spin-valley locking mechanism discussed in the main text.

Turning to technical questions, the construction of the self-energy in Eq. (9) demands the knowledge of a relevant set of eigenvectors of the Hamiltonian. That self-energy is defined as the sum over all the occupied states in the electronic bands, but in practice one has to impose some kind of truncation when carrying out the calculation. In this respect, we have retained the first 51 valence bands in the self-consistent resolution.

Moreover, we have adopted a mixed representation of the electronic states by performing a Fourier transform passing to momenta $\mathbf{k}$ in the superlattice of the twisted trilayer. That is, we build the electron operators as

$$a_{n,i,\sigma} = \frac{1}{\sqrt{N_c}} \sum_{\mathbf{k}\in BZ} a(\mathbf{k})_{i,\sigma} e^{i\mathbf{k}\cdot(\mathbf{r}_i + \mathbf{R}_n)} \tag{10}$$

where $\mathbf{r}_i$ are the coordinates of the carbon atoms in the supercell, $\mathbf{R}_n$ are lattice vectors in the superlattice of the twisted trilayer, and the sum is over momenta in the Brillouin zone of the superlattice (N_c is the number of unit cells). In practice, we compute the self-energy taking a grid with 192 momenta (plus the Gamma point) covering the Brillouin zone. We have checked that such a content of states is safe to capture the relevant symmetry-breaking patterns of twisted trilayer graphene, as well as to obtain a sensible description of its low-energy bands.

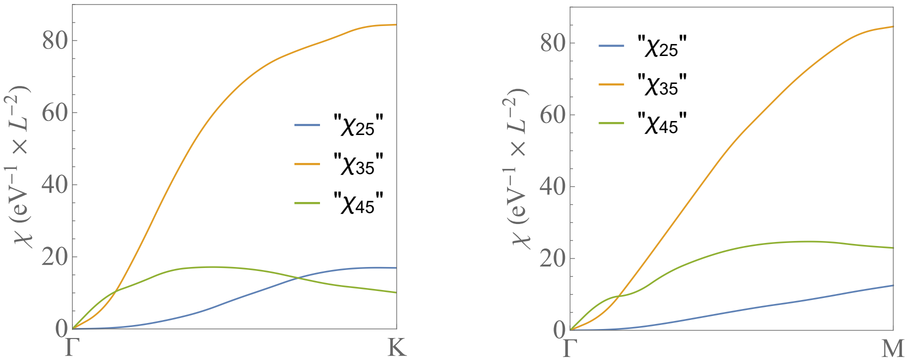

(a) (b)

Supplementary Figure 3: Evolution along the ΓK (a) and the ΓM line (b) of the particle-hole susceptibility computed with the bands of the interacting theory represented in Fig. 3 of the main text. The plot shows the partial contributions χ_{ij} between the three flat conduction bands ($i = 2, 3, 4$) and the flat valence band ($j = 5$). The susceptibility is measured in units of eV$^{-1} \times L^{-2}$, where L is the lattice constant of the moiré superlattice.

SUPPLEMENTARY NOTE III.
INTERNAL SCREENING AND DIELECTRIC CONSTANT

An important question in the discussion of the electronic properties is the determination of the dielectric constant ϵ to be used for twisted trilayer graphene. The magnitude of that quantity depends mainly on the internal screening of the Coulomb interaction, which becomes rather intense as a consequence of the reduced bandwidth of the lowest-energy valence and conduction bands. A good estimate of the dielectric constant can be obtained from the dielectric function $\epsilon(\mathbf{q}, \omega)$, which can be computed in the RPA for the two-dimensional Coulomb interaction as

$$\epsilon(\mathbf{q}, \omega) = 1 + \frac{e^2}{2\epsilon_0 |\mathbf{q}|} \chi(\mathbf{q}, \omega) \tag{11}$$

where $\chi(\mathbf{q}, \omega)$ stands for the particle-hole susceptibility. We are going to be interested in the effects of internal screening at length scales of the order of the size of the supercell of the twisted trilayer, as the interaction is already screened at long distances by the presence of metallic gates in our model. Then, we can estimate the magnitude of the dielectric constant from the values of the dielectric function $\epsilon(\mathbf{q}, 0)$ at momenta $\mathbf{q}$ of the order of the inverse of the lattice constant of the superlattice.

In order to make a reliable estimate of the internal screening, we compute the dielectric function with the bands of the interacting theory represented in Fig. 3 of the main text. This is a suitable situation, as there is a gap separating the flat valence and conduction bands around the Fermi level. Then, we will be able to check the consistency of our estimates by comparing them with the actual value of the dielectric constant used to obtain the bands in the mentioned figure. The largest contributions to $\epsilon(\mathbf{q}, 0)$ come indeed from the four flat bands around the Fermi level. We can approximate the susceptibility by considering particle-hole excitations between the three flat conduction bands (numbered as $i = 2, 3, 4$) and the flat valence band below the Fermi level (numbered as $j = 5$). These lead to the partial contributions shown as χ_{ij} in Fig. 3 along the directions ΓK and ΓM.

We have for the dielectric function computed for instance at the large momentum $\mathbf{Q}_K$ of the K point in the moiré Brillouin zone

$$\epsilon(\mathbf{Q}_K, 0) = 1 + \frac{e^2}{2\epsilon_0 |\mathbf{Q}_K|} \chi(\mathbf{Q}_K, 0) \tag{12}$$

$$= 1 + \frac{3}{8\pi\epsilon_0} e^2 \, L \, \chi(\mathbf{Q}_K, 0) \tag{13}$$

where L stands for the lattice constant of the moiré superlattice. We take $e^2/\epsilon_0 \approx 17.7$ eV nm and the length $L \approx 8.46$ nm for a twisted trilayer belonging to the sequence of commensurate superlattices with $\theta \approx 1.61°$. The particle-hole susceptibility can be obtained by adding the different contributions from Fig. 3. Taking into account the spin degeneracy, we get the estimate

$$\epsilon(\mathbf{Q}_K, 0) \approx 55 \tag{14}$$

Interestingly, we obtain a very similar magnitude if we carry out the estimate at the M point of the moiré Brillouin zone, taking again the values for the particle-hole susceptibility from the curves shown in Fig. 3.

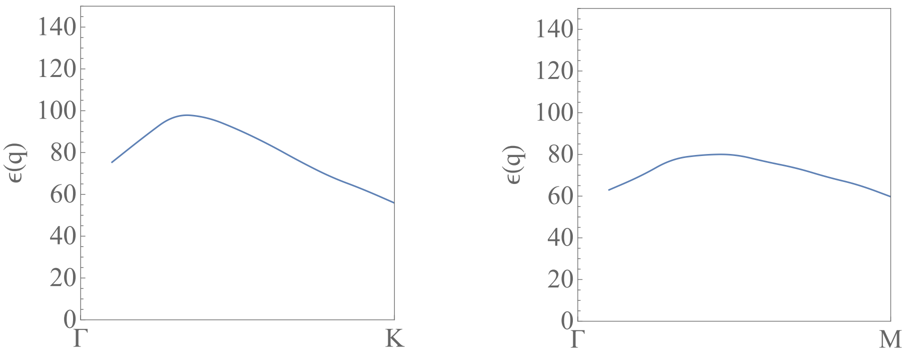

(a) (b)

Supplementary Figure 4: Evolution along the ΓK (a) and the ΓM line (b) of the dielectric function computed with the bands of the interacting theory represented in Fig. 3 of the main text.

The full shape of the dielectric function, computed with the procedure just outlined, is represented along the ΓK and ΓM directions in Fig. 4. As already mentioned, we take the Coulomb interaction in our calculations with a screening length $\xi = 10$ nm from the presence of metallic gates. This means that we can encode the internal screening in the form of an effective dielectric constant by looking at momenta not much smaller than the inverse of the lattice constant of the superlattice. As seen in Figs. 4(a)-(b), the behavior of the dielectric function is rather smooth towards the boundary of the moiré Brillouin zone. The values of $\epsilon(\mathbf{q}, 0)$ at $\mathbf{Q}_K$ and $\mathbf{Q}_M$ are actually lower bounds, so it makes sense to take them as conservative estimates of the dielectric constant. Moreover, they turn out to be only slightly larger than the value used to obtain the bands in Fig. 3 of the main text, which shows the consistency of our determination of the internal screening in the model.

We remark that the effect of screening from the dielectric environment can be included in the above computation, but making almost no difference in the final result, as long as the value of the dielectric constant in (14) is much larger than any typical dielectric constant ϵ_{env} of the substrate. That is, one can introduce ϵ_{env} instead of ϵ_0 in the above derivation, but this would reduce correspondingly the effect of the particle-hole susceptibility, leading to the cancellation of ϵ_{env} in the final expression for the effective Coulomb potential $e^2/2\epsilon|\mathbf{q}|$, with the value of ϵ already given by (14).

SUPPLEMENTARY NOTE IV.
ORDER PARAMETERS AND PHASE DIAGRAM OF TWISTED TRILAYER GRAPHENE

In the self-consistent Hartree-Fock resolution, an important role is played by the matrix elements

$$h_{ij}^{(\sigma)} = {\sum_a}' \phi_{a\sigma}(\boldsymbol{r}_i)\phi^*_{a\sigma}(\boldsymbol{r}_j) \; . \tag{15}$$

where $\phi_{a\sigma}(\boldsymbol{r}_i)$ stand for the eigenvectors of the different states labeled by a and the spin σ, and depending on the atomic positions $\boldsymbol{r}_i$. The prime means that the sum is to be carried over the occupied levels. These matrix elements become also very useful in the definition of the order parameters for broken-symmetry phases. This is due to the fact that they coincide with the values of the equal-time propagator for the electron operators $a_{i\sigma}$. It can be actually shown that

$$\langle a^+_{j\sigma}(t)a_{i\sigma}(t)\rangle = {\sum_a}' \phi_{a\sigma}(\boldsymbol{r}_i)\phi^*_{a\sigma}(\boldsymbol{r}_j) \tag{16}$$

This means that different charge densities as well as hopping amplitudes can be written in terms of $h_{ij}^{(\sigma)}$.

The main charge instability corresponds indeed to a mismatch in the charge densities for the two different sublattices A and B in each graphene layer. This leads to chiral symmetry breaking, with the opening of a gap between the low-energy Dirac cones at the charge neutrality point. Locally, the order parameter is given by the charge asymmetry

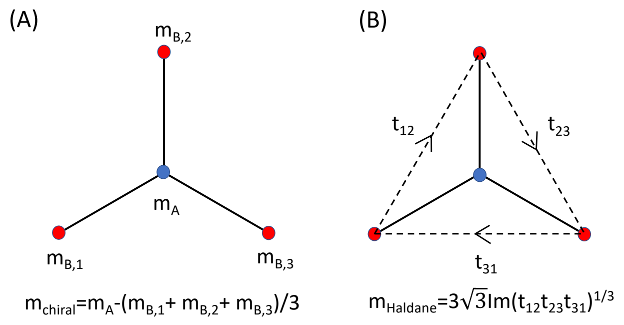

Supplementary Figure 5: Schematic definition of the two main symmetry breaking patterns opening a gap in the honeycomb lattice: (A) chiral symmetry breaking leading to a Dirac mass (where we have replaced h_{ii} defined in the text by m), (B) time-reversal symmetry breaking leading to the Haldane mass (where we have replaced h_{ij} defined in the text by t_{ij}).

between each carbon atom and its nearest neighbors, as represented in Fig. 5. Globally, the order parameter is defined by the quantity

$$C^{(\sigma)} = \sum_{i\in A} h_{ii}^{(\sigma)} - \sum_{i\in B} h_{ii}^{(\sigma)} \tag{17}$$

The other way to open a gap between the low-energy Dirac cones consists in producing an effective magnetic flux at each atomic site, which can be assessed by adding the phases of the hopping matrix elements between nearest neighbors i_1, i_2 and i_3 of each atom i, as represented in Fig. 5. The effective flux leads to time-reversal and parity symmetry breaking, conferring a so-called Haldane mass to the low-energy Dirac fermions. The order parameter for this broken-symmetry phase is given by

$$P_+^{(\sigma)} = \mathrm{Im}\left(\sum_{i\in A} \left(h_{i_1 i_2}^{(\sigma)} h_{i_2 i_3}^{(\sigma)} h_{i_3 i_1}^{(\sigma)} \right)^{\frac{1}{3}} + \sum_{i\in B} \left(h_{i_1 i_2}^{(\sigma)} h_{i_2 i_3}^{(\sigma)} h_{i_3 i_1}^{(\sigma)} \right)^{\frac{1}{3}} \right) \tag{18}$$

where the nearest neighbors i_1, i_2 and i_3 are always taken with a definite orientation.

Furthermore, there is also the possibility of having an effective magnetic flux but preserving parity, which is realized by attaching opposite fluxes at atoms belonging to different sublattices A and B. The order parameter characterizing this phase is

$$P_-^{(\sigma)} = \mathrm{Im}\left(\sum_{i\in A} \left(h_{i_1 i_2}^{(\sigma)} h_{i_2 i_3}^{(\sigma)} h_{i_3 i_1}^{(\sigma)} \right)^{\frac{1}{3}} - \sum_{i\in B} \left(h_{i_1 i_2}^{(\sigma)} h_{i_2 i_3}^{(\sigma)} h_{i_3 i_1}^{(\sigma)} \right)^{\frac{1}{3}} \right) \tag{19}$$

In the continuum theory of Dirac fermions, it can be shown that this breakdown of symmetry translates into the generation of a term proportional to the identity in pseudospin space. This does not open a gap in the Dirac cones at the K point, but the shift in the energy of the cones becomes different in the two valleys of the electron system. The main effect corresponds therefore to spin-selective valley symmetry breaking, which is indeed a ubiquitous feature in graphene multilayers away from the charge neutrality point.

The evolution of the order parameters (17)-(19) can be studied as the strength of the extended Coulomb interaction is varied. The most interesting instance corresponds to a filling fraction of 2 holes per moiré unit cell. Then the dominant pattern of symmetry breaking corresponds to $P_-^{(\sigma)}$, while $P_+^{(\sigma)}$ and $C^{(\sigma)}$ open up beyond a certain interaction strength. This is illustrated in the phase diagram shown in Fig. 6, which is the result of applying the self-consistent Hartree-Fock approximation for the screening length ξ = 10nm. With our estimate of the dielectric constant (14), we have $e^2/4\pi\epsilon \approx 0.22$ eV$\times a$ (where a is the C-C distance). This places the interaction in a regime where, apart from spin-selective valley symmetry breaking, there is also a breakdown of time-reversal symmetry leading to a Chern insulator phase. The origin of this phase lies in the fact that, at the filling fraction of 2-hole doping, valley symmetry breaking for each spin channel sets the Fermi level at the vertices of the Dirac cones of the lower valley. The Dirac nodes may then be destabilized for a sufficiently strong interaction, mainly due to the appearance of the Haldane

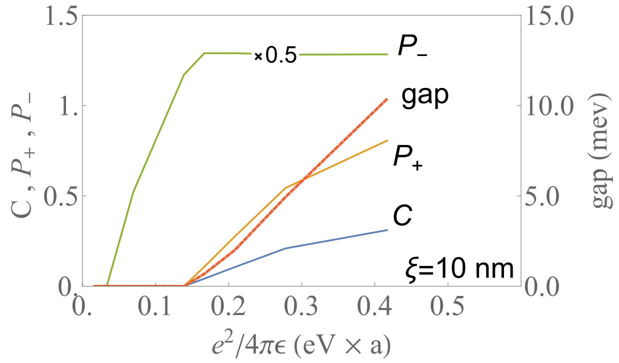

Supplementary Figure 6: Phase diagram showing the different order parameters of symmetry breaking at a filling fraction of 2 holes per moiré unit cell in twisted trilayer graphene, obtained by means of the self-consistent Hartree-Fock approximation with screening length $\xi = 10$ nm for the extended Coulomb potential. The interaction strength is measured in units of eV times the C-C distance a.

mass. This explains the opening of the gap at 2-hole doping, which is the relevant instance discussed in the main text.

We finally comment on the possibility of having order parameters which reflect in the form of microscope structure in the twisted trilayer, at the level of the hexagonal lattices of the layers. In this respect, we have checked that the fluxes that make up $P_+^{(\sigma)}$ and $P_-^{(\sigma)}$ do not show any microscopic pattern in the graphene lattices, for all interactions strengths ranging from small to large values of the dielectric constant ϵ. A particular instance is illustrated at the end of this Supplemental Material, where one can see that the fluxes in the microscopic triangular loops have a smooth envelop across the unit cell of the moiré superlattice.

However, it is more interesting the case of the order parameter for the so-called K-intervalley coherence, which has been discussed at length in Ref. [6] for magic-angle twisted bilayer graphene. This order parameter takes the form of a magnetization density wave at the wave vector K of graphene, with circulating currents along the hexagonal rings combining into a typical kekulé pattern which triples the graphene unit cell. In our microscopic approach, we can characterize such an order parameter by measuring the flux enclosed in the six-fold rings made of consecutive nearest-neighbors sites i_1 to i_6 in the graphene lattice (with a fixed orientation). We then define the quantity

$$P_{\rm KIVC}^{(\sigma)}(\boldsymbol{r}_i) = h_{i_1 i_2}^{(\sigma)} h_{i_2 i_3}^{(\sigma)} h_{i_3 i_4}^{(\sigma)} h_{i_4 i_5}^{(\sigma)} h_{i_5 i_6}^{(\sigma)} h_{i_6 i_1}^{(\sigma)} \qquad (20)$$

This allows us to capture the signature of K-intervalley coherence by looking for microscopic structure in the angle $\theta_{\rm KIVC}$ given by

$$P_{\rm KIVC}^{(\sigma)} = |P_{\rm KIVC}^{(\sigma)}| e^{i\theta_{\rm KIVC}} \qquad (21)$$

We have computed $\theta_{\rm KIVC}$ across the supercell of the twisted trilayer, looking for a definite pattern at the microscopic scale. However, we have only found negative evidence in that respect, for strong coupling of the Coulomb interaction as well as in the regime of the twisted trilayer considered in the paper. This can be seen in the plots shown in Fig. 7, which represent the values of $\theta_{\rm KIVC}$ at the hexagonal rings of each layer for two different values of the dielectric constant $\epsilon = 12$ and 48. The envelop of the angles in the supercell gives rise to a smooth surface in all cases, showing the absence of K-intervalley coherence in the twisted trilayer.

The above negative result points at a marked difference between the behavior of twisted bilayer and twisted trilayer graphene at the magic angle, as we have checked that a similar microscopic Hartree-Fock approach applied to the twisted bilayer (with in-plane relaxation) leads indeed to signatures of K-intervalley coherence for values of the dielectric constant as large as $\epsilon \sim 40$. This deviation between the two systems may come from the different type of relevant relaxation (in-plane versus out-of-plane) which one needs to consider in each case. This prevents from assuming a simple decoupling of twisted trilayer graphene as a system of twisted bilayer plus a single graphene layer. As shown in the first section of this Supplemental Material, the out-of-plane corrugation leads to important modifications in the shape of the flat bands of twisted trilayer graphene, inducing a significant particle-hole asymmetry which has a large impact in the symmetry breaking properties of the system.

SUPPLEMENTARY NOTE V.

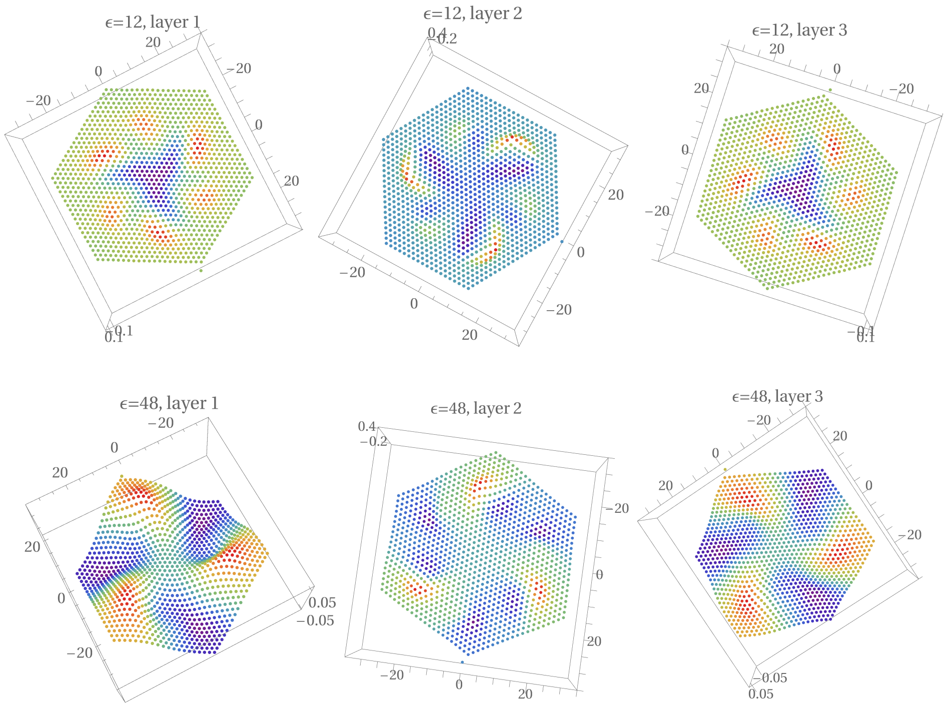

Supplementary Figure 7: Plot of the fluxes enclosed in the hexagonal rings of each layer of twisted trilayer graphene, for two different values of the dielectric constant $\epsilon = 12$ (upper row) and 48 (lower row). Darker red (blue) color corresponds to higher positive (lower negative) values of the flux.

SEMICLASSICAL THEORY OF THE HALL DENSITY

In this Section, we will analyze the Hall density, accessible in typical transport experiments [7]. Within a semiclassical theory, electrons move on trajectories of constant energy. For small magnetic fields, these trajectories are not altered and we will thus use the energy contours of the flat-band dispersion obtained from the self-consistent Hartree-Fock calculations. For the first valence band we use the filling factor $\nu = -2$, for the second valence band we use the filling factor $\nu = -2.8$. In both cases, the dielectric constant is set to $\epsilon = 48$.

The contours can be divided into closed and open trajectories. Closed trajectories can be approximated by circular, elliptic or trigonal warped curves. Open trajectories shall be characterized by van Hove singularities. In the following, we will obtain analytical formulas for all these situations.

Supplementary Note V.A.: Closed trajectories

For closed trajectories, we calculate the conductivity via the Chambers' formula

$$\sigma_{ij} = \frac{g_s g_v e^2}{(2\pi)^2} \int d^2k v_i(\boldsymbol{k}) \int_{-\infty}^{0} dt' v_j(\boldsymbol{k}(t')) e^{t'/\tau} \left(-\frac{\partial f(E)}{\partial E} \right) \ . \tag{22}$$

By virtue of the Lorentz force rule, this can be transformed into the following expression[8]:

$$\sigma_{ij} = \frac{g_s g_v}{(2\pi)^2} \frac{e^3 B}{\hbar^2} \int_0^T dt v_i(t) \int_{-\infty}^{t} dt' v_j(t') e^{(t'-t)/\tau} \tag{23}$$

The velocities $v_i(t)$ are obtained from the semiclassical equations,

$$\dot{\boldsymbol{r}} = \mathbf{v}(\boldsymbol{k}) = \frac{1}{\hbar} \partial_{\boldsymbol{k}} \epsilon_{\boldsymbol{k}} \ , \tag{24}$$

$$\dot{\boldsymbol{k}} = -\frac{e}{\hbar} \left[\mathbf{E} + \mathbf{v}(\boldsymbol{k}) \times \mathbf{B} \right] \ . \tag{25}$$

We assume the magnetic field perpendicular to the plane and the electric field in x-direction, i.e., $\mathbf{B} = B\mathbf{e}_z$ and $\mathbf{E} = E\mathbf{e}_x$. The equations of motion in the plane can be integrated and yield

$$\boldsymbol{r}(t) = -\frac{\hbar}{eB}\mathbf{e}_z \times \boldsymbol{k}(t) - \frac{E}{B}\mathbf{e}_y t \ . \tag{26}$$

The drift velocity $\mathbf{v}_D = -\frac{E}{B}\mathbf{e}_y$ is in fact the velocity of the frame of reference in which the electric field vanishes.[9] With $\widetilde{\epsilon}_{\boldsymbol{k}} = \epsilon_{\boldsymbol{k}} - \hbar\boldsymbol{k}\cdot\mathbf{v}_D$, we can thus combine the equations of motion to the following compact form where only the magnetic field enters:

$$\dot{\boldsymbol{k}} = -\frac{e}{\hbar^2}\partial_{\boldsymbol{k}}\widetilde{\epsilon}_{\boldsymbol{k}} \times \mathbf{B} \ , \tag{27}$$

In the following, we assume a small electric field and neglect the drift term.

Closed orbits are periodic in T and can thus be expanded into a Fourier series. We can therefore set

$$k_i(t) = \sum_\nu \kappa_\nu^i e^{i\nu\omega_c t} \ , \tag{28}$$

where we introduced the two components $i = x, y$ and the cyclotron frequency $\omega_c = 2\pi/T$. We now obtain the velocities $v_i(t)$ by differentiating Eq. (26) and with Chambers' formula, this finally yields

$$\sigma = \frac{g_s g_v}{2\pi}\frac{e^2\tau}{m_c}\sum_{\nu>0}\frac{\nu^2}{1+(\nu\omega_c\tau)^2}\begin{pmatrix} |\kappa_\nu^y|^2 & -\operatorname{Re}\left[\kappa_\nu^x\kappa_{-\nu}^y(1+i\nu\omega_c\tau)\right] \\ -\operatorname{Re}\left[\kappa_\nu^x\kappa_{-\nu}^y(1-i\nu\omega_c\tau)\right] & |\kappa_\nu^x|^2 \end{pmatrix} . \tag{29}$$

Since κ_ν^x is independent of B, this generally proves the Onsager relation $\sigma_{xy}(B) = \sigma_{yx}(-B)$. To be more explicit, we will now discuss isotropic and elliptic models.

Supplementary Note V.A.1.: Circular and elliptic curves

For the general isotropic dispersion $\epsilon_{\boldsymbol{k}} = \alpha|k|^\xi$, the Fermi wave number is given by $k_F = (\mu/\alpha)^{1/\xi}$ with μ the chemical potential. This yields the circular trajectories $k_x(t) = k_F\cos(\omega_c t)$ and $k_y(t) = k_F\sin(\omega_c t)$ with the cyclotron frequency $\omega_c = \frac{eB}{m_c}$ and cyclotron mass $m_c = \frac{\hbar^2}{\xi\alpha}k_F^{2-\xi}$. Consistently, this is the same result as obtained from the general definition $m_c = \frac{\hbar^2}{2\pi}\frac{\partial A}{\partial\mu}$ with $A = \pi k_F^2$ denoting the area that is enclosed by the cyclotron orbit. With $(g_s g_v/4)k_F^2 = \pi n$, where g_s and g_v denote spin and valley (or other) degeneracies, this then gives the final result

$$\sigma = \frac{\sigma_{xx}}{1+\tilde{a}^2}\begin{pmatrix} 1 & \tilde{a} \\ -\tilde{a} & 1 \end{pmatrix} , \tag{30}$$

where the longitudinal conductivity is given by $\sigma_{xx} = e^2 n\tau/m_c$ and $\tilde{a} = \omega_c\tau$. With the resistivity tensor $\rho = \sigma^{-1}$, we get for all isotropic dispersion relations the universal Hall density

$$n_H = -\left[e\frac{d\rho_{xy}}{dB}\right]^{-1} = n \ . \tag{31}$$

The universal result also holds for an elliptic dispersion with $\epsilon_{\boldsymbol{k}} = \alpha_x k_x^2 + \alpha_y k_y^2$ and $k_{F,i} = \sqrt{\mu/\alpha_i}$. With $k_x(t) = k_{F,x}\cos(\omega_c t)$, $k_y(t) = k_{F,y}\sin(\omega_c t)$, $A = \pi k_{F,x}k_{F,y}$ and $\omega_c = \frac{eB}{m_c}$, we have $m_c = \frac{\hbar^2}{2\sqrt{\alpha_x\alpha_y}}$ and

$$\sigma = \frac{\sigma_{xx}}{1+\tilde{a}^2}\begin{pmatrix} \sqrt{\alpha_x/\alpha_y} & \tilde{a} \\ -\tilde{a} & \sqrt{\alpha_y/\alpha_x} \end{pmatrix} , \tag{32}$$

where $\sigma_{xx} = \frac{e^2 n\tau}{m_c}$. With Eq. (31), we again obtain the universal expression $n_H = n$ for the Hall density independent of the band parameters.

Supplementary Note V.A.2.: Trigonal warped trajectories

For trigonal warped Fermi-surfaces, there are deviations from the universal result. However, in a perturbative treatment the first non-vanishing term is quadratic in the expansion parameter $\epsilon \sim k_F a$. This suggests that general closed orbits will lead to a Hall density close to the universal result, i.e., $n = n_H$. This shall be discussed below.

To proceed analytically, let us discuss single-layer graphene in the trigonal warped regime. Graphene is characterized by the energy dispersion $\epsilon_{\boldsymbol{k}} = \pm t|\Phi_{\boldsymbol{k}}|$ where $\Phi_{\boldsymbol{k}} = \sum_\delta e^{i\boldsymbol{k}\cdot\delta}$ with the three nearest-neighbor vectors $\delta_1 = a(1,0)$, $\delta_2 = a(-1/2, \sqrt{3}/2)$, and $\delta_3 = a(-1/2, -\sqrt{3}/2)$ as well as $t = -2.7$ the hopping matrix element. With the Jacobi-Anger expansion, the structure factor can also be written as $\Phi_{\boldsymbol{k}} = 3\sum_n J_{-1+3n}(ka)e^{i(-1+3n)\phi}$ [10]. To lowest order in the lattice effect, we then have the following expression for the Fermi surface in polar coordinates:

$$k'_F(\phi) = k_F\left(1 + \epsilon\cos(3\phi) + \frac{\epsilon^2}{4}\left[11 + 5\cos(6\phi)\right]\right) \tag{33}$$

The enclosed area is still given by $A = \pi k_F^2$ with $E_F = \hbar v_F k_F$ and $v_F = \frac{3}{2}at$. We further introduced the trigonal warping parameter $\epsilon = \frac{k_F a}{4}$.

With $\mathbf{v} = -\frac{\hbar}{eB}\mathbf{e}_z \times \dot{\boldsymbol{k}}$, we have $\partial_{\boldsymbol{k}}\epsilon_{\boldsymbol{k}} = \frac{\hbar^2}{eB}\left[k\dot{\phi}\mathbf{e}_k - \dot{k}\mathbf{e}_\phi\right]$. With the dimensionless parameter $\bar{k} = k/k_F$, this gives the following set of differential equations:

$$\bar{k}\dot{\phi} = \omega_c\left[1 - 2\bar{k}\epsilon\cos(3\phi) - \frac{3}{4}(\bar{k}\epsilon)^2\left[7 + \cos(6\phi)\right]\right] \tag{34}$$

$$\dot{\bar{k}} = -3\omega_c\bar{k}\epsilon\sin(3\phi) - \frac{3}{2}\omega_c(\bar{k}\epsilon)^2\sin(6\phi) \tag{35}$$

To second order, the solution thus reads $\bar{k}(t) = 1 + \epsilon\cos(3\omega_c t) + \frac{\epsilon^2}{4}(17 - \cos(6\omega_c t))$ and $\phi(t) = \omega_c t - \epsilon\sin(3\omega_c t) + \epsilon^2(-12\omega_c t + \frac{1}{2}\sin(6\omega_c t))$. For the cartesian coordinates, we then have $k_x(t) = k'_F(\phi(t))\cos(\phi(t))$ and $k_y(t) = k'_F(\phi(t))\sin(\phi(t))$. Note that for $k'_F(\phi(t))$ only the expansion of ϕ up to first order is needed in order to be consistent.

We can now again discuss the response in the presence of a magnetic field via the Chambers' formula. For the explicit solution of the trigonal warped graphene regime, we get

$$\sigma = \frac{e^2 n\tau}{m_c}\frac{1}{(1+(\omega_c\tau)^2)(1+4(\omega_c\tau)^2)}\begin{pmatrix} 1 + 12\epsilon^2 + 4(1+9\epsilon^2)(\omega_c\tau)^2 & \omega_c\tau\left[1 + 4(1+6\epsilon^2)(\omega_c\tau)^2\right] \\ -\omega_c\tau\left[1 + 4(1+6\epsilon^2)(\omega_c\tau)^2\right] & 1 + 12\epsilon^2 + 4(1+9\epsilon^2)(\omega_c\tau)^2 \end{pmatrix}. \tag{36}$$

The Hall number is usually defined by

$$\frac{1}{n_H} = \frac{e}{B}\frac{\sigma_{xy}}{\sigma_{xx}\sigma_{yy} + \sigma_{xy}^2}. \tag{37}$$

This gives the final result

$$\frac{n_H}{n} = 1 + \left[6 + \frac{18}{1 + 4(\omega_c\tau)^2}\right]\epsilon^2. \tag{38}$$

In the clean limit $\tau \to \infty$, this simplifies to $n_H = n(1+6\epsilon^2)$ and in the low-field limit $\omega_c \to 0$, we have $n_H = n(1+24\epsilon^2)$. In both cases, there is thus a slight increase of the Hall number due to the acceleration around the corners of the deformed Brillouin zone.

The Hall number is also sometimes defined by

$$\frac{1}{n_H} = e\frac{d}{dB}\frac{\sigma_{xy}}{\sigma_{xx}\sigma_{yy} + \sigma_{xy}^2}. \tag{39}$$

This gives for the Hall density

$$\frac{n_H}{n} = 1 + \frac{24\left[1 - (\omega_c\tau)^2 + 4(\omega_c\tau)^4\right]}{(1+4(\omega_c\tau)^2)^2}\epsilon^2, \tag{40}$$

which is a slightly different expression than above. However, in the clean limit, this again simplifies to $n_H = n(1+6\epsilon^2)$ and we also have $n_H = n(1 + 24\epsilon^2)$ for the low-field limit as before.

To conclude, there is no linear correction in ϵ to the Hall density. The deviations from the universal result $n_H = n$ should thus be small and negligible. We shall, therefore, approximate $n_H = n$ for all closed trajectories.

Supplementary Note V.B.: Trajectories close to van Hove singularities

To discuss the semiclassical motion of electrons close to van Hove singularities, a well-defined regularization procedure is needed since the orbits are unbounded for a continuum theory. Therefore, we will not use the Chambers' formula, but start from the macroscopic equations of motion for the current density. The general response theory in the presence of an in-plane electric field $\mathbf{E}$ and a perpendicular magnetic field $\mathbf{B}$ then reads

$$\partial_t \mathbf{j} = \chi \mathbf{E} + \frac{e}{m_c} \bar{\mathbf{j}} \times \mathbf{B} - \mathbf{j}/\tau \; . \tag{41}$$

Above, we introduced the current-current response function χ in the dc-limit and the "average" current density $\bar{\mathbf{j}}$ which will both be discussed below. We also introduced the inverse relaxation time $\eta = \tau^{-1}$ and the cyclotron mass is defined by[9]

$$m_c = \frac{\hbar^2}{2\pi} \frac{\partial A}{\partial \mu} \; , \tag{42}$$

where A denotes the area that is enclosed by the cyclotron orbit. Within this formalism, the above results for the isotropic and elliptic models can be obtained. Here, we will outline the specific case of a hyperbolic model.

Supplementary Note V.B.1.: Drude response around a saddle-point

The van Hove singularity shall be described by the saddle-point dispersion $\epsilon_{\boldsymbol{k}} = -\alpha_- k_x^2 + \alpha_+ k_y^2$. The so-called Drude response can entirely be obtained from the band structure and for $T = 0$ at the chemical potential μ, it is defined by

$$\chi_{ij} = \frac{g_s g_v e^2}{(2\pi\hbar)^2} \int d^2k (\nabla \epsilon_{\boldsymbol{k}})_i (\nabla \epsilon_{\boldsymbol{k}})_j \delta(\mu - \epsilon_{\boldsymbol{k}}) \; . \tag{43}$$

As we have assumed the principle axes to be along the x- and y-direction, $\chi_{ij} \propto \delta_{ij}$.

The above integral can be performed by first eliminating the δ-function via the polar integration. For the radial integration, the following integrals are needed:

$$\mathcal{I}_\pm(\Lambda, \gamma) = \int_1^{\Lambda^2} dx \sqrt{\frac{x-1}{\gamma x + 1}}^{\pm 1} \tag{44}$$

This gives for $\mu = \pm|\mu|$ the final expression

$$\chi_\pm = \frac{g_s g_v}{(2\pi)^2} \frac{e^2}{\hbar^2} \frac{4\tilde{\mu}_\pm}{(\alpha_+ + \alpha_-)} \begin{pmatrix} \alpha_-^2 \mathcal{I}_\pm(\tilde{\Lambda}_\pm, \gamma_\pm) & 0 \\ 0 & \alpha_+^2 \mathcal{I}_\mp(\tilde{\Lambda}_\pm, \gamma_\pm) \end{pmatrix} \; , \tag{45}$$

with $\gamma_\pm = \alpha_\mp/\alpha_\pm$, $\tilde{\mu}_\pm = |\mu|/\alpha_\pm$, and $\tilde{\Lambda}_\pm = \Lambda/\sqrt{\tilde{\mu}_\pm}$ where Λ denotes the wavenumber cutoff. In the following, we will only discuss the response due to electron doping with $\mu > 0$ and set $\gamma = \gamma_+$.

At the neutrality point, the susceptibility is proportional to Λ^2 and we will discuss the difference $\delta\chi = \chi_+ - \chi_{\mu=0}$. To leading order, we have

$$\delta\chi = \frac{g_s g_v}{(2\pi)^2} \frac{e^2}{\hbar^2} 2\mu \begin{pmatrix} -\sqrt{\gamma} \ln \frac{\alpha\Lambda^2}{\mu} & 0 \\ 0 & \sqrt{\gamma}^{-1} \ln \frac{\alpha\Lambda^2}{\mu} \end{pmatrix} \; , \tag{46}$$

where $\alpha = \frac{2\alpha_+\alpha_-}{\alpha_+ + \alpha_-}$. The area relative to the one of $\mu = 0$ is given by $A = 4\frac{\mu}{\sqrt{\alpha_+\alpha_-}} \ln \frac{4\alpha_-\Lambda^2}{\mu}$. Therefore, we get to leading order in Λ the cyclotron mass $m_c = 4\frac{\hbar^2}{2\pi\sqrt{\alpha_+\alpha_-}} \ln \frac{4\alpha_-\Lambda^2}{\mu}$. With $n = \frac{g_s g_v}{(2\pi)^2} A$, this yields

$$\frac{m_c}{e^2} \delta\chi = \frac{n}{\pi} \begin{pmatrix} -\sqrt{\gamma} \ln \frac{\alpha\Lambda^2}{\mu} & 0 \\ 0 & \sqrt{\gamma}^{-1} \ln \frac{\alpha\Lambda^2}{\mu} \end{pmatrix} \; . \tag{47}$$

Supplementary Note V.B.2.: Magnetic response around a saddle-point

Let us now include the magnetic field. A magnetic field does not break rotational invariance and for an anisotropic system, the field couples to the average velocity $v^2 = v_x v_y$. For an elliptic dispersion, this yields the universal results $n_H = n$ as mentioned above.

In the case of a saddle-point, however, we also have to keep track of the negative sign and we have to couple to the positive mean velocity $v^2 = -v_x v_y$. From the cartesian velocities $v_i = \hbar^{-1}\partial_{k_i}\epsilon_{\boldsymbol{k}}$ and $\mathbf{j} = -en\mathbf{v}$, we thus set $\bar{\mathbf{j}} = (j_x/\sqrt{\gamma}, -\sqrt{\gamma}j_y)$. The hyperbolic response with respect to $\mu = 0$ gives then rise to the following conductivity tensor:

$$\sigma = \frac{\tau}{1-\tilde{a}^2}\begin{pmatrix} \delta\chi_1 & -\tilde{a}\sqrt{\gamma}\delta\chi_2 \\ -\tilde{a}\delta\chi_1/\sqrt{\gamma} & \delta\chi_2 \end{pmatrix} , \tag{48}$$

where we used $\delta\chi = \mathrm{diag}(\delta\chi_1, \delta\chi_2)$ of Eq. (46). The resistivity tensor thus reads

$$\rho = \frac{\eta}{\delta\chi_1\delta\chi_2}\begin{pmatrix} \delta\chi_2 & \tilde{a}\sqrt{\gamma}\delta\chi_2 \\ \tilde{a}\delta\chi_1/\sqrt{\gamma} & \delta\chi_1 \end{pmatrix} . \tag{49}$$

Therefore, we get for the Hall density the final result

$$n_H = -\left[e\frac{d\rho_{xy}}{dB}\right]^{-1} = \frac{n}{\pi}\ln\frac{\alpha\Lambda^2}{\mu} . \tag{50}$$

There is a logarithmic divergence for $\mu \to 0$ which has been discussed also in the context of a tight-binding model.[8] However, for extended van Hove singularities there is also a possible divergence in the limit $\alpha \to 0$ which is independent of μ.

SUPPLEMENTARY NOTE VI.
NUMERICAL DISCUSSION OF THE HALL DENSITY

We will now numerically discuss the Hall density starting with the first valence band. For hole doping up to $\nu \approx -1.8$, no gap has developed yet and the transport is dominated by hole-doping. Close to the neutrality point, all semiclassical trajectories are closed and we have $n_H \sim n$. This "universal behavior" is, however, modified by the presence of two van Hove singularities which shall be modeled by Eq. (50). At the filling factor $\nu \sim -1.8$, we observe a merging of the three van Hove singularities at the Γ-point to form a so-called higher-order van Hove singularity.[11] Beyond that point, a gap is formed due to time-reversal symmetry breaking leading to electronic transport with universal behavior. This is consistent with the experimentally observed Hall reset at $\nu = -2$.

To discuss the Hall density of the second valance band, we start from the symmetric transport model, i.e., half of the band is dominated by electron transport and the other half by hole transport. This is justified by noting that close to the band edges, the trajectories are all closed. Again, this "universal behavior" is modified by the presence of two van Hove singularities which is also modeled by Eq. (50).

Before we outline the fitting procedure, let us recall that we find a prominent valley symmetry breaking for each spin channel which reduces the inherent C_6-symmetry to a C_3-symmetry. This symmetry is also reflected in the van Hove singularities which are always composed of saddle points that come in triplets. The positions of the van Hove singularities usually lie on the six ΓM directions. However, for $\nu \lesssim -1.8$, the two triplets lie on the same three ΓM directions which enforces the valley-symmetry broken state and induces a gap. In Fig. 8, the contour plots of the two valence bands are shown, highlighting the energy contours at the two van Hove energies, respectively. The initial discretization of the Brillouin zone was given by 20 k-points between the two K points. With the moiré supercell lattice constant a_M, the wave numbers k_x and k_y are thus in units of $\frac{1}{20}\frac{4\pi}{3a_M}$.

The expression for the Hall density around a van Hove singularity depends on the parameters α and Λ which shall now be determined. Due to numerical errors, the C_3-symmetry regarding the three-fold saddle points is not exact even though the appearance in the contour plot suggests this approximate symmetry. We thus choose to fit the saddle points along the principle axis by the general dispersion $\epsilon_{\boldsymbol{k}} = -\alpha_- k_-^2 + \alpha_+ k_+^2$ which is closest to a parabola with positive and negative mass. In Fig. 8, we indicate and numerate the saddle points that were used in the fitting process.

We now fit the van Hove singularities along the principle axes as indicated in Fig. 9. Obviously, this procedure could be improved by considering curved trajectories obeying the C_3-symmetry, however, we checked that the overall result hardly depends on it. By fitting the dispersion along the principle axes, there is also sometimes another scaling

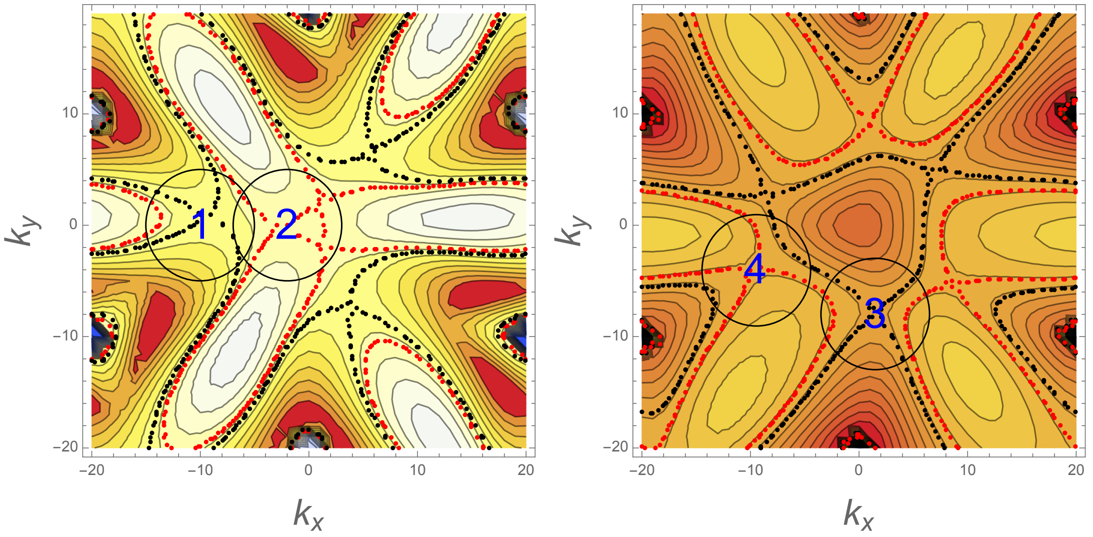

Supplementary Figure 8: Self-consistent energy dispersion of the first (left) and second (right) valence band for $\nu = -2$ and $\nu = -2.8$, respectively. The black and red dotted lines indicate the energy contour at the van Hove energies. The wave numbers are in units of $\frac{1}{20}\frac{4\pi}{3a_M}$ with a_M the moiré lattice constant.

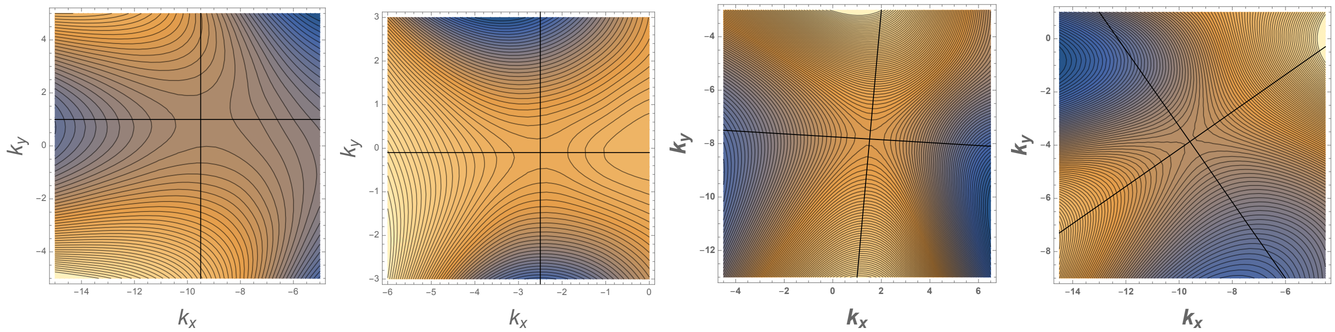

Supplementary Figure 9: Zoom-in of the van Hove singularities 1, 2, 3, 4 (from left to right). Also shown are the approximated principle axes along which the parameters of the saddle points are fitted. The wave numbers are in units of $\frac{1}{20}\frac{4\pi}{3a_M}$ with a_M the moiré lattice constant.

factor to be considered as we usually parametrize our curves by either k_x or k_y. The explicit comparison between the dispersion and the fitting result as function of these scaled wave numbers k_- and k_+ is shown in Fig. 10. The results are listed in Table I.

What is left is the determination of the band cutoff Λ. This is done by demanding continuity with the universal regime $n_H = n$ at the cross-over chemical potential μ^* corresponding to the crossover density n^*:

$$\Lambda^2 = \frac{\mu^*}{\alpha} e^{\pi} \tag{51}$$

For the crossover density $|n^* - n_{vH}| = \Delta n$, we set $\Delta n = 0.15$. For doping levels between two of the van Hove singularities, i.e., in the range $|n_{vH,1} - n_{vH,2}| - 2\Delta n$, we choose a linear interpolation of the two logarithmic singularities. Note that by construction, there appears a discontinuity at $n_{vH} + \Delta n$ for the second van Hove singularity due to the change from hole to electron transport. This abrupt change should be smeared out in more realistic models.

Finite temperature effects as well as possible disorder effects are included by substituting $|\mu| \to |\mu| + k_B T$ that smears out the logarithmic singularity. This makes the cutoff parameter dependent of the temperature and/or disorder. In Table I, we report the results for $T = 0$, 70 mK, 1 K. Let us note that $T = 70$ mK is the temperature used in the experiments of Ref. 7, however, we obtain the best fit for $T = 1$ K which suggests that there is considerable disorder in the sample without gate voltage.

Let us finally comment on the contribution of the Dirac cone that has been neglected in our analysis, so far. Due to mirror reflection symmetry, the flat bands and the Dirac cone decouple and can be treated separately. Dirac cones

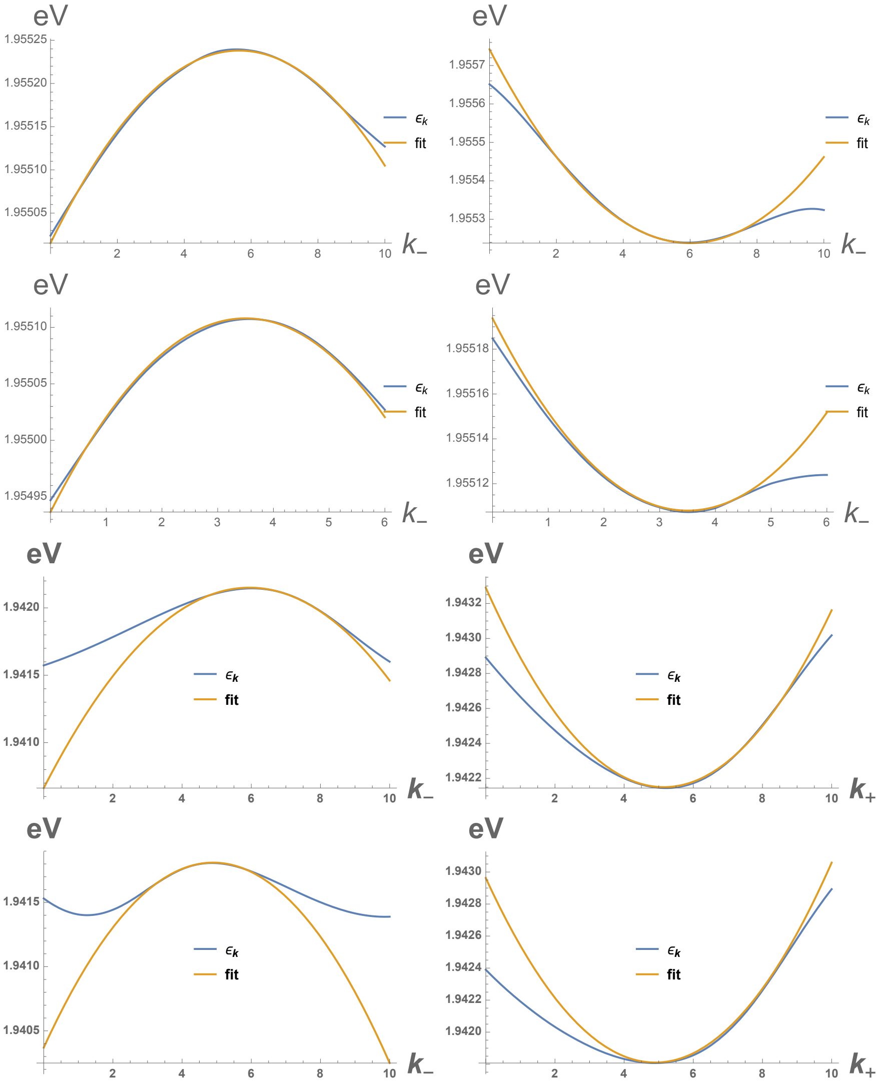

Supplementary Figure 10: Fitting results of the van Hove singularities 1, 2, 3, 4 (from top to bottom) for the negative (left) and positive (right) inverse mass.

i	ϵ_{vH}	n_{vH}	α_-	α_+	α	$\Lambda_{0\text{K}}$	$\Lambda_{70\text{mK}}$	$\Lambda_{1\text{K}}$
1	1.9552	-1.12	0.0334	0.0668	0.0447	4.83	5.15	8.25
2	1.9551	-1.51	0.0668	0.0334	0.0447	4.20	4.56	7.90
3	1.9422	-2.73	0.1998	0.2032	0.2015	3.88	3.97	4.99
4	1.9418	-3.21	0.1922	0.1538	0.1709	3.57	3.68	4.94

Supplementary Table 1: Energy of the van Hove singularities ϵ_{vH} (in eV), the corresponding filling number n_{vH}, and the fitting parameters $\alpha_\pm$ in units of meV a_M^2 defining the saddle point dispersion $\epsilon_{\boldsymbol{k}} = -\alpha_- k_-^2 + \alpha_+ k_+^2$. We also list the scale that enters the expression of the Hall density, $\alpha = \frac{2\alpha_+\alpha_-}{\alpha_+ + \alpha_-}$ (in meV a_M^2), and the band cutoff Λ_T (in a_M^{-1}) for $T = 0$, 70 mK, 1 K.

lead to circular trajectories due to their conical nature and thus lead again to universal behavior $n_H = n$ (assuming hole doping). However, the hole doping is only a fraction of the doping of the moiré supercell and can usually be neglected. Only, for $\nu \approx -4$, the contribution should be measurable and in fact, a small offset of n_H at $\nu = -4$ is seen in the experiments of Ref. 7 which we attribute to the Dirac cone contribution.

SUPPLEMENTARY NOTE VII.
PAIRING INSTABILITIES

Pairing instabilities can be studied by looking for singularities in the so-called BCS vertex, when incoming and outgoing electrons have total momentum equal to zero. For this purpose, we may collect the most divergent contributions in this channel, which leads to the iteration of particle-particle diagrams encoded in the diagrammatic equation shown in Fig. 11. The particle-particle loop at the right-hand-side of the equation involves an integration in momentum space, that can be parametrized in terms of the components $k_\parallel$ and $k_\perp$ which are parallel and normal, respectively, to the contour lines of constant energy. Alternatively, we can make a change of variables to the energy ε of the contour lines and the angle θ along them. Then, the self-consistent equation for the BCS vertex V becomes

$$V(\theta,\theta';\omega) = V_0(\theta,\theta') - \frac{1}{(2\pi)^2}\int_0^\Lambda d\varepsilon \int_0^{2\pi} d\theta'' \frac{\partial k_\perp}{\partial \varepsilon}\frac{\partial k_\parallel}{\partial \theta''} V_0(\theta,\theta'')\frac{1}{\varepsilon-\omega}V(\theta'',\theta';\omega) \tag{52}$$

where θ, θ' are the angles of the respective momenta of the spin-up incoming and outgoing electrons and ω is the sum of the frequencies of the modes in the pair.

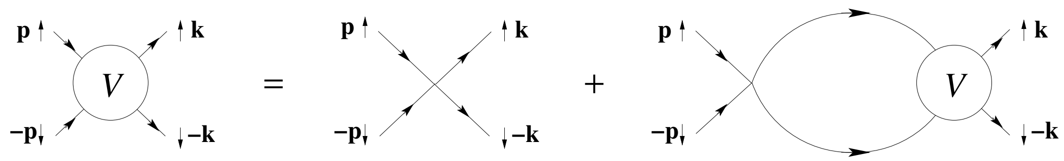

Supplementary Figure 11: Self-consistent diagrammatic equation for the BCS vertex V encoding the iteration of Cooper-pair scattering.

Eq. (52) can be cast in a more compact form by making the change of variables

$$\widehat{V}(\theta,\theta';\omega) = \sqrt{\frac{1}{2\pi}\frac{\partial k_\perp(\theta)}{\partial \varepsilon}\frac{\partial k_\parallel(\theta)}{\partial \theta}}\sqrt{\frac{1}{2\pi}\frac{\partial k_\perp(\theta')}{\partial \varepsilon}\frac{\partial k_\parallel(\theta')}{\partial \theta'}}V(\theta,\theta';\omega) \tag{53}$$

After that, we can take the derivative with respect to the high-energy cutoff Λ and apply the self-consistency at the right-hand-side of the equation, to end up in the scaling equation

$$\Lambda\frac{\partial \widehat{V}(\theta,\theta';\omega)}{\partial \Lambda} = -\frac{1}{2\pi}\int_0^{2\pi} d\theta'' \widehat{V}(\theta,\theta'';\omega)\widehat{V}(\theta'',\theta';\omega) \tag{54}$$

In Eq. (54) it is implicit that the BCS vertex must be actually a function of the ratio ω/Λ. Then, the scaling equation can be also used to find the behavior of the vertex in the low-energy limit $\omega \to 0$.

The analysis of Eq. (54) is facilitated by expanding the vertex in a set of orthogonal modes $\Psi_m^{(\gamma)}(\theta)$ corresponding to the different representations γ of the point symmetry group,

$$\widehat{V}(\theta,\theta';\omega) = \sum_{\gamma,m,n} V_{m,n}^{(\gamma)}(\omega)\Psi_m^{(\gamma)}(\theta)\Psi_n^{(\gamma)}(\theta') \tag{55}$$

We arrive then at the set of equations

$$\omega\frac{\partial V_{m,n}^{(\gamma)}}{\partial \omega} = \sum_s V_{m,s}^{(\gamma)}V_{s,n}^{(\gamma)} \tag{56}$$

where we have assumed that the vertex must depend on the combination ω/Λ.

In this framework, a pairing instability arises when any of the eigenvalues in the expansion (55) has a negative value $V^{(\gamma)}(\Lambda_0) < 0$ at the high-energy cutoff. Then, the solution of (56) leads to a divergent flow given by

$$V^{(\gamma)}(\omega) = \frac{V^{(\gamma)}(\Lambda_0)}{1 + V^{(\gamma)}(\Lambda_0)\log\left(\frac{\Lambda_0}{\omega}\right)} \tag{57}$$

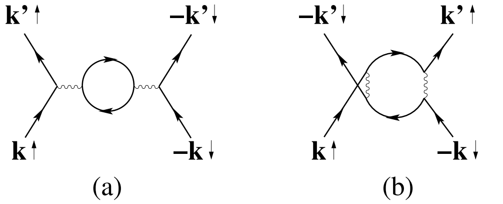

Supplementary Figure 12: Second-order diagrams contributing to the unrenormalized BCS vertex.

In the flow towards the low-energy limit $\omega \to 0$, a singularity is reached at a critical frequency

$$\omega_c \approx \Lambda_0 \exp\{-1/|V^{(\gamma)}(\Lambda_0)|\} \tag{58}$$

which sets the scale of the superconducting instability.

In practice, one has to start with a sensible representation of the vertex $V(\theta,\theta')$ at the high-energy cutoff Λ_0. This can be obtained by performing a sum of particle-hole contributions, building on the original diagrams which were considered in the seminal work by Kohn and Luttinger. Usually, one resorts to iterate in the particle-hole scattering shown in Figs. 12(a)-(b) [12]. In our case, an important difference with respect to the discussion carried out for the Hubbard model is that the interaction is mediated by the extended Coulomb potential $v(\boldsymbol{r})$, as we are dealing with all the atoms in the unit cell of the moiré superlattice. Then, the sum of RPA and ladder contributions leads to an expression for the vertex

$$V(\theta,\theta';\Lambda_0) = \frac{v(\mathbf{k}-\mathbf{k}')}{1+v(\mathbf{k}-\mathbf{k}')\chi_{\rm ph}(\mathbf{k}-\mathbf{k}')} + \frac{v^2(\mathbf{Q})\widetilde{\chi}_{\rm ph}(\mathbf{k}+\mathbf{k}')}{1-v(\mathbf{Q})\widetilde{\chi}_{\rm ph}(\mathbf{k}+\mathbf{k}')} \tag{59}$$

where $\chi_{\rm ph}$ ($\widetilde{\chi}_{\rm ph}$) stands for the susceptibility in the series of bubble (ladder) diagrams. The interaction $v(\mathbf{Q})$ is a function of the momentum transfer $\mathbf{Q}$ which depends on the sum of the momenta $\mathbf{k},\mathbf{k}'$ of incoming and outgoing electrons as well as on the momentum of the internal loop. In Eq. (59), the sum of RPA diagrams leads to screening of the interaction, making its contribution less relevant, while it is the sum of ladder diagrams encoded in the second term what may enhance potential pairing instabilities.

Once we compute the BCS vertex according to Eq. (59), the last stage of the analysis is the evaluation of the different coefficients in the expansion (55) at the high-energy cutoff. This can be easily made using the orthogonality of the modes, so that

$$V^{(\gamma)}_{m,n}(\Lambda_0) = \int_0^{2\pi} d\theta \int_0^{2\pi} d\theta' \; \widehat{V}(\theta,\theta';\Lambda_0)\Psi^{(\gamma)}_m(\theta)\Psi^{(\gamma)}_n(\theta') \tag{60}$$

This is the approach we have followed to determine the different eigenvalues for the BCS vertex, applying in particular the convolution (60) with a large set of harmonics to capture the modulations along the energy contour lines of the second valence band.

The results of this decomposition of the BCS vertex in harmonics may differ significantly, depending on whether the Fermi line displays the triangular patches found above the van Hove singularity at $\nu \approx -2.8$ or it has evolved into elliptical shape below that filling fraction. These two different possibilities are illustrated in Fig. 13. The first regime has been discussed at $\nu = -2.4$ in the main text, and we analyze here the two instances shown in Figs. 13(a)-(b) for filling fraction $\nu = -2.8$ and $\nu = -3.6$.

The Fermi line shown in Fig. 13(a) has an approximate C_{3v} symmetry, so that the eigenmodes in the expansion of the BCS vertex can be assorted in irreducible representations of that group. The first terms in the series of eigenvalues an respective eigenvectors can be seen in Table II. We find that there are two irreps with relatively prominent negative eigenvalues, although slightly smaller in absolute value than those obtained in the expansion at $\nu = -2.4$. We can introduce these values into Eq. (58) to estimate T_c. Taking $\Lambda_0 \approx 1.5$ meV, we obtain an estimate of $T_c \sim 1$ K. Although the order of magnitude is similar to that found for T_c at $\nu = -2.4$, the absolute value of the dominant negative coupling is smaller at $\nu = -2.8$, implying that the critical temperature has to be necessarily smaller at such a larger hole doping.

Turning to the Fermi line in Fig. 13(b), the elliptical shape has an approximate C_{2v} symmetry, which means that the different eigenvalues of the BCS vertex correspond to one-dimensional representations. The different couplings for the particular case shown in Fig. 13(b) are listed in Table III. We observe that there are several negative eigenvalues, which imply that the elliptic Fermi line still may support a pairing instability. The critical energy scale has to be obtained according to Eq. (58), bearing in mind that Λ_0 must be a symmetric cutoff dictated by the effective

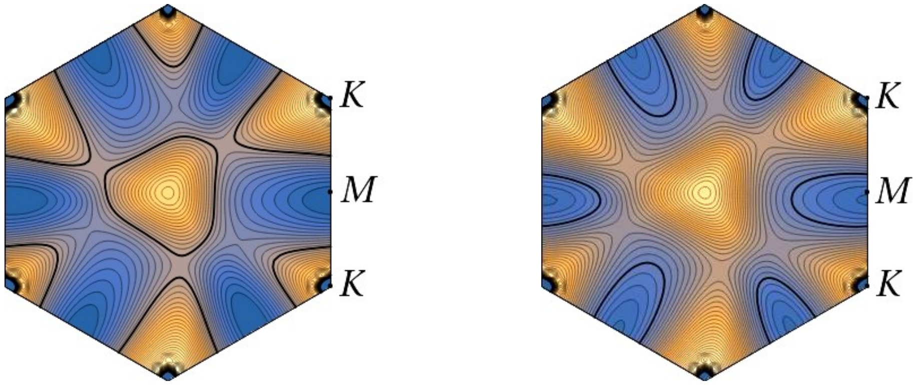

(a) (b)

Supplementary Figure 13: Energy contour maps of the second valence band (for spin-up projection) in the Brillouin zone of twisted trilayer graphene at twist angle $\theta \approx 1.61°$, computed in a self-consistent Hartree-Fock approximation with dielectric constant $\epsilon = 48$ and filling fraction of 2.8 holes (a) and 3.6 holes (b) per moiré unit cell. The thick contours stand for the Fermi lines. Contiguous contour lines differ by a constant step of 0.1 meV, from lower energies in blue to higher energies in light color.

Eigenvalue λ	harmonics	Irr. Rep.
2.31	1	
1.25<br>1.24	$\{\cos(\phi), \sin(\phi)\}$	E
0.46	$\cos(3\phi)$	A_1
−0.30<br>−0.29	$\{\cos(4\phi), \sin(4\phi)\}$	E
−0.29	$\sin(3\phi)$	A_2
0.27<br>0.26	$\{\cos(4\phi), \sin(4\phi)\}$	E
0.15	$\cos(6\phi)$	A_1
0.15<br>0.13	$\{\cos(5\phi), \sin(5\phi)\}$	E

Supplementary Table 2: Eigenvalues of the Cooper-pair vertex with largest magnitude and dominant harmonics, grouped according to the irreducible representations of the approximate C_{3v} symmetry, for the Fermi line shown in Fig. 13(a). The modes $\{\cos(4\phi), \sin(4\phi)\}$ appear twice in the list, as they only denote the dominant harmonic, but they actually represent different eigenvectors.

Eigenvalue λ	harmonics	Irr. Rep.
0.85	1	
−0.25	$\sin(\phi)$	B_2
−0.11	$\cos(\phi)$	B_1
0.08	$\cos(3\phi)$	B_1
−0.06	$\sin(2\phi)$	A_2
−0.04	$\cos(2\phi)$	A_1

Supplementary Table 3: Eigenvalues of the Cooper-pair vertex with largest magnitude and dominant harmonics along the elliptic Fermi lines shown in Fig. 13(b) for filling fraction of 3.6 holes per moiré unit cell.

bandwidth, here constrained by the proximity of the Fermi line to the bottom of the band. We estimate $\Lambda_0 \approx 0.4$ meV which leads, for the dominant negative coupling in Table III, to a critical temperature $T_c \sim 0.1$ K.

We arrive at the general conclusion that the Kohn-Luttinger instability is stronger in the regime where twisted trilayer graphene develops the rather regular triangular Fermi lines observed in the second valence band, in the range within filling fractions $\nu \approx -2$ and $\nu \approx -2.8$. The pairing instability then looses strength for larger hole doping, as a consequence of having smaller pairing modulation as well as much smaller energy range for the scattering of Cooper pairs, which produces a substantial decrease in the critical temperature when approaching the bottom of the band.

SUPPLEMENTARY NOTE VIII. EFFECTIVE SPIN-ORBIT COUPLING, ISING SUPERCONDUCTIVITY AND VIOLATION OF THE PAULI-LIMIT

The spin-selective valley symmetry breaking is driven by the emerging flux that is generated by the imaginary part of the next-nearest neighbour hopping $t_X^{(\ell)}$, with the two sublattices $X = A, B$ and the layer $\ell = 1, 2, 3$. This flux has opposite sign for the two sublattices and thus valley symmetry breaking in each spin channel is the dominate order parameter related to $(t_A^{(\ell)} - t_B^{(\ell)})/2$. Nevertheless, there is also a net Haldane flux that leads to a time-reversal symmetry broken gap related to $(t_A^{(\ell)} + t_B^{(\ell)})/2$.

The graphs shown in Fig. 14 display the results for one spin projection. The values are reversed for the other spin projection such that time-reversal symmetry is only broken for each spin sector individually. Combining the two spin-channels, time-reversal symmetry is restored just as it is the case in the Kane-Mele model,[13] only with an effective intrinsic spin-orbit coupling. This leads to a pinning of the spin polarization perpendicular to the layer.

To make the discussion quantitative, let us set the maximal imaginary tunnel-matrix element of layer 2 and of sublattice A/B as $3t_A^{(2)} = 0.001$ eV and $3t_B^{(2)} = -0.0015$ eV, respectively, as shown in Fig. 14. The energy scale for the spin gap $\Delta = 2\sqrt{3}3t$ is thus given by $\Delta = 2\sqrt{3} \times 0.25$meV ~ 1 meV.

So far, the initial Hamiltonian had no spin-orbit coupling such that the spin-polarization of the Cooper pairs would be arbitrary. However, due to the bare intrinsic spin-orbit coupling of single-layer graphene, the spin-degeneracy is broken and leads to an out-of-plane spin-polarization. The effective, renormalized intrinsic spin-orbit coupling thus also leads to out-of-plane polarized spin-singlet Cooper-pairs as was already discussed the context of graphene by Kane and Mele.[13]

Because of the out-of-plane polarization, these spin-states are unaffected by an in-plane magnetic field unless the magnetic field energy surpasses the pinning energy. In this case, the singlet of the Cooper-pair is first rotated parallel to the field and then broken up due to the energy gained from the magnetic susceptibility, characterized by the Pauli-limit which corresponds to an energy less than the pinning energy. This leads to a violation of the Pauli-limit by a factor of 2-3 as argued in the main text.

SUPPLEMENTARY REFERENCES

[1] P. Moon and M. Koshino, Phys. Rev. B **87**, 205404 (2013).
[2] A. N. Kolmogorov and V. H. Crespi, Phys. Rev. Lett. **85**, 4727 (2000).
[3] A. N. Kolmogorov and V. H. Crespi, Phys. Rev. B **71**, 235415 (2005).
[4] R. E. Throckmorton and O. Vafek, Phys. Rev. B **86**, 115447 (2012).
[5] A. L. Fetter and J. D. Walecka, *Quantum Theory of Many-Particle Systems* (McGraw-Hill, New York, 1971).
[6] N. Bultinck, E. Khalaf, S. Liu, S. Chatterjee, A. Vishwanath, and M. P. Zaletel, Phys. Rev. X **10**, 031034 (2020).
[7] J. M. Park, Y. Cao, K. Watanabe, T. Taniguchi, and P. Jarillo-Herrero, Nature **590**, 249 (2021).
[8] A. V. Maharaj, I. Esterlis, Y. Zhang, B. J. Ramshaw, and S. A. Kivelson, Phys. Rev. B **96**, 045132 (2017).
[9] N. W. Ashcroft and N. D. Mermin, *Solid State Physics* (Saunders College Publishing, 1976).
[10] G. Gómez-Santos and T. Stauber, Phys. Rev. Lett. **106**, 045504 (2011).
[11] J. González and T. Stauber, unpublished.
[12] D. J. Scalapino, E. Loh, and J. E. Hirsch, Phys. Rev. B **35**, 6694 (1987).
[13] C. L. Kane and E. J. Mele, Phys. Rev. Lett. **95**, 226801 (2005).

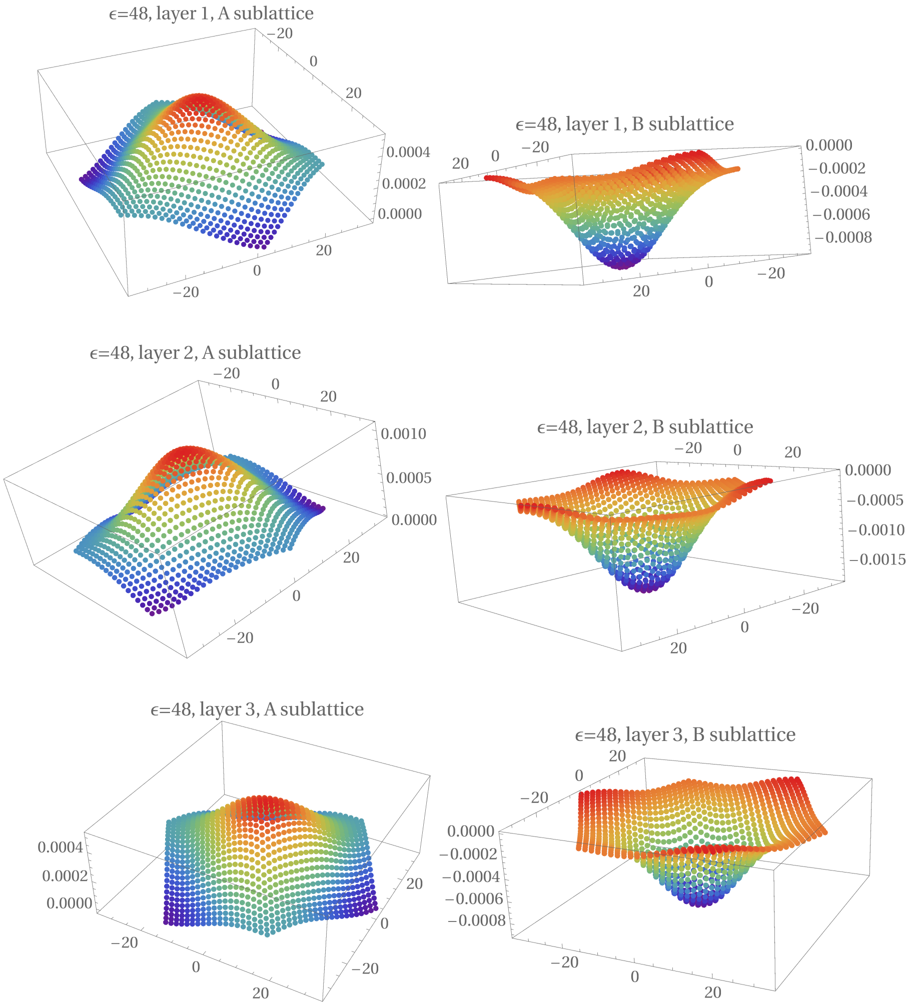

Supplementary Figure 14: Plot on the moiré cell of the imaginary part of the next-nearest neighbour hopping $t_X^{(\ell)}$ with sublattice index $X = A, B$ and layer index $\ell = 1, 2, 3$ for one spin-projection. The panels show $3t_X^{(\ell)}$ and the absolute value is concentrated around the AA-stacked region. The vertical scale is in units of eV.
